# Factors Associated With Hypertension in Ethiopia: A Systematic Review, Meta‐Analysis, and Quantitative Framework for Comparative Association

**DOI:** 10.1155/ijhy/7268884

**Published:** 2026-11-01

**Authors:** Martins Nweke, Julian D. Pillay

**Affiliations:** ^1^ Department of Basic Medical Science Faculty of Health Sciences Durban University of Technology Durban South Africa dut.ac.za

**Keywords:** associated factors, comparative association analysis, Ethiopia, hypertension, meta-analysis, risk clustering, systematic review

## Abstract

**Background:**

Hypertension is a leading cause of cardiovascular morbidity and mortality worldwide and represents a growing public health challenge in Ethiopia. Multiple demographic, metabolic, behavioral, and socioeconomic factors contribute to its development; however, the relative importance of these factors remains unclear.

**Objective:**

To synthesize evidence on factors associated with hypertension in Ethiopia and compare the strength and consistency of these associations.

**Methods:**

A systematic review was conducted following the PRISMA guidelines, with searches of PubMed/MEDLINE, Scopus, EMBASE, Web of Science, the Cumulative Index for Nursing and Allied Health Literature (CINAHL), and African Journals Online (SABINET) from inception to March 2026. Observational studies reporting factors associated with hypertension in adults in Ethiopia were included. We performed a random‐effects meta‐analysis for factors with sufficient homogeneity, whereas other variables were synthesized narratively. A quantitative framework based on risk weight estimates was used to compare the strength and consistency of the associations and explore risk clustering; the findings were interpreted as indicative rather than causal.

**Results:**

Forty‐one studies, predominantly cross‐sectional. Older age was strongly associated with hypertension (pooled OR 2.90, 95% CI: 2.07–4.07). Obesity and being overweight were consistently associated with increased odds (pooled OR ≈ 2.0–3.5). Diabetes showed a markedly elevated association (pooled OR = 11.47, 95% CI: 1.11–118.23), with wide confidence intervals. Behavioral factors, including alcohol use and physical inactivity, showed moderate but inconsistent associations, whereas socioeconomic factors were context‐dependent. Risk weight estimates indicated that diabetes (Rw = 2.5) and older age (Rw = 1.8) had the most consistent association with the outcome.

**Conclusion:**

In Ethiopia, hypertension is strongly associated with metabolic factors, particularly age and diabetes, as well as with variable behavioral and socioeconomic influences. These findings support integrated prevention strategies that target high‐risk groups, defined here as groups with comparatively stronger observed associations with hypertension rather than prospectively established risk.

## 1. Introduction

Hypertension is a major global public health problem and one of the leading modifiable risk factors for cardiovascular disease, stroke, chronic kidney disease, and premature death. Globally, approximately 1.28 billion adults aged 30–79 years are estimated to have hypertension, with a substantial proportion living in low‐ and middle‐income countries (LMICs) [1]. Despite the availability of effective antihypertensive therapies and established prevention strategies, hypertension remains poorly detected and inadequately controlled in several settings [2]. The burden is particularly concerning in sub‐Saharan Africa, where rapid urbanization, population aging, dietary changes, physical inactivity, obesity, and other social and behavioral transitions contribute to an increasing burden of cardiovascular risk [3].

Ethiopia is experiencing a similar epidemiological transition as that of India. Hypertension is increasingly recognized as an important contributor to cardiovascular morbidity and mortality; however, awareness, treatment, and control remain suboptimal [4–6]. Previous studies conducted in different Ethiopian populations have reported considerable variations in the prevalence of hypertension, reflecting differences in age structure, study setting, urbanization, sampling methods, and diagnostic approaches [7–10]. Beyond these contextual differences, hypertension in Ethiopia is influenced by a broad range of demographic, behavioral, dietary, anthropometric, metabolic, socioeconomic, and clinical factors. Older age, obesity, diabetes, family history of hypertension, physical inactivity, alcohol consumption, dietary practices, and other factors have been reported in individual studies [11–18]. However, the magnitude and consistency of these associations vary across different populations and settings.

Several systematic reviews and meta‐analyses have synthesized evidence from Ethiopia. Earlier reviews primarily estimated the pooled prevalence of hypertension and examined selected determinants of hypertension [19, 20]. Recently, Ayalew et al. synthesized evidence from 22 studies involving 14,202 participants and reported pooled associations for several factors, including age, sex, family history of hypertension, physical inactivity, obesity, khat chewing, and salt consumption [21]. Similarly, Kassie et al. conducted a systematic review and meta‐analysis specifically on undiagnosed hypertension, including 10 cross‐sectional studies, and identified older age, elevated BMI, family history of hypertension, and comorbid diabetes as important associated factors [22]. The existing literature has established that several individual factors are associated with hypertension in Ethiopia, and the present review does not claim that such associations have not been previously synthesized.

However, important gaps remain in the interpretation of these findings. First, previous reviews have generally evaluated individual associated factors separately, making it difficult to compare the relative strength and consistency of associations across a wide spectrum of demographic, metabolic, behavioral, dietary, socioeconomic, and clinical factors. Second, the evidence base has continued to expand, with additional primary studies published since the searches were undertaken in earlier reviews. Third, the previous review of undiagnosed hypertension addressed a narrower outcome than the broader spectrum of hypertension considered in the present review [22]. Consequently, an updated and broader synthesis is needed to determine whether the associations reported across individual Ethiopian studies are consistently observed and how their magnitude compares across different exposure domains.

Accordingly, this systematic review and meta‐analysis aimed to synthesize the expanded evidence on factors associated with hypertension among adults in Ethiopia, evaluate the strength and consistency of these associations across major domains, and explore patterns of co‐occurring factors within the available evidence in Ethiopia. In contrast to previous reviews that primarily synthesized prevalence and selected individual associations, the present review places particular emphasis on the comparative interpretation of the magnitude and consistency of associations across multiple domains and uses an exploratory quantitative framework to characterize recurring combinations of factors. This approach is intended to provide a more integrated understanding of heterogeneous evidence and identify patterns that may inform population‐level prevention strategies and guide future longitudinal research.

### 1.1. Review Questions

1. What factors are associated with hypertension in Ethiopia?
2. What is the relative strength and consistency of the observed associations when assessed using a quantitative comparative association framework?
3. What patterns of co‐occurrence among associated factors can be identified to provide a multilevel understanding of hypertension in Ethiopia?

### 1.2. Review Objectives

1. To systematically synthesize the evidence on the factors associated with hypertension in Ethiopia.
2. To compare the relative magnitude and consistency of the observed associations using a quantitative comparative association framework.
3. To explore patterns of co‐occurrence among associated factors to support a structured interpretation of the determinants of hypertension in Ethiopia.

### 1.3. Definition of Key Terms

Association: A statistical relationship between an exposure or characteristic and hypertension, as observed in the included observational studies. An observed association does not necessarily imply a causal relationship [23].

Risk refers to the likelihood that a defined health outcome will occur within a population, such as the probability of developing lung cancer among individuals exposed to asbestos. In contrast, etiology concerns the underlying causes or origins of a disease. In this study, the term “risk” was used in a broader population‐health sense to describe the relative burden or likelihood of hypertension associated with particular characteristics or combinations of characteristics, rather than to imply prospective risk or causality. Because the evidence base is predominantly cross‐sectional, the observed relationships are interpreted primarily as associations and cannot establish temporality or causal effects [12]. Accordingly, references to “higher‐risk” or “lower‐risk” groups in this review denote groups with comparatively greater or lower levels of observed hypertension‐associated characteristics rather than groups for whom future hypertension risk has been prospectively demonstrated.

Factors associated with hypertension: Demographic, behavioral, metabolic, socioeconomic, or clinical characteristics that showed a statistical relationship with hypertension in the included observational studies. These factors may be modifiable or nonmodifiable, and their identification does not imply causality [14].

Causality: A relationship in which an exposure contributes to the occurrence of an outcome. Establishing causality requires consideration of temporality and other epidemiological criteria. Because most studies included in this review were cross‐sectional, this review did not establish causal relationships [12].

Comparative association stratification: In this review, this refers to the comparative assessment of the relative magnitude and consistency of associations across factors using the quantitative framework described in the Methods section. The framework is intended for population‐level interpretation and prioritization of observed associations rather than individual clinical predictions [15, 16].

### 1.4. Conceptual Framework for the Interpretation of Associations

Hypertension is a multifactorial condition influenced by a combination of demographic, metabolic, behavioral, socioeconomic, and environmental characteristics [14]. Therefore, the conceptual framework for this review was used to organize and interpret the observed associations across these domains rather than to establish causal relationships. The framework was informed by the multicausal perspective of Rothman’s sufficient‐component cause model, which conceptualizes disease occurrence as involving multiple component factors and potentially different combinations of contributing conditions [17]. In the present review, this perspective was used only as a conceptual model to support the classification and integrated interpretation of factors that co‐occur in the available Ethiopian evidence. It was not used to infer that the observed factors constitute the component causes of hypertension or that particular combinations are sufficient to produce the outcome. Bradford Hill’s viewpoints were also considered as interpretive principles when assessing the strength and consistency of observed associations across studies [18]. However, these viewpoints have not been applied as formal causal assessment tools. In particular, temporality could not be adequately evaluated because the included studies were predominantly cross‐sectional. Consequently, the framework was used to support a structured interpretation of patterns, consistency, and heterogeneity in the observed associations, rather than to establish causal relationships. Accordingly, the conceptual framework supports the organization of factors into demographic, metabolic, behavioral, and socioeconomic domains and facilitates the examination of how these factors may co‐occur within the available evidence. Therefore, any identified patterns should be interpreted as patterns of observed association rather than evidence of causal pathways, joint causal effects, or individual‐level risk prediction.

## 2. Methods

### 2.1. Protocol and Registration

This study was a systematic review with a meta‐analysis. The conduct and reporting of the review adhered to the Preferred Reporting Items for Systematic Reviews and Meta‐Analyses (2020) guidelines to ensure transparency, reproducibility, and methodological rigor [21]. The review methodology was informed by the Joanna Briggs Institute (JBI) guidelines for systematic reviews of etiology and risk [13]. The review protocol was registered with PROSPERO (registration number: CRD420261281264). Following the peer review and iterative refinement of the manuscript, minor amendments were made to the original protocol. These included clarification of outcome definitions, refinement of inclusion and exclusion criteria, and adjustment of the analytical framework to emphasize the interpretation of associations rather than causal inferences. These modifications did not alter the overall objectives of the review but improved its methodological clarity and alignment with the available evidence.

### 2.2. Eligibility Criteria

This review applied the PECODTL framework (population, exposure, comparison, outcome, design, time frame, and language) to guide study selection and ensure a structured and consistent approach to eligibility and data extraction [22].

### 2.3. Inclusion Criteria

#### 2.3.1. Population (P)

Studies conducted in Ethiopia involving adults (≥ 18 years), including individuals with and without hypertension, were eligible, irrespective of sex or other demographic characteristics of the participants.

#### 2.3.2. Exposure (E)

This review considered the exposures examined in relation to hypertension in primary studies, which were broadly grouped as follows:

• Biological and metabolic factors (e.g., age, sex, obesity, diabetes, kidney disease, HIV status, and antiretroviral therapy)
• Behavioral factors (e.g., smoking, alcohol consumption, physical inactivity, and dietary patterns, including salt intake and fruit/vegetable consumption)
• Socioeconomic and psychosocial factors (e.g., education, occupation, income, marital status, and stress)
• Only exposures evaluated in relation to hypertension outcomes were included in this study.

#### 2.3.3. Comparison (C)

Eligible studies compared individuals with hypertension to those without hypertension or reported measures of association across exposure categories (e.g., high vs. low‐exposure groups).

#### 2.3.4. Outcome (O)

The primary outcome was hypertension, which was defined as follows:

Hypertension was defined primarily according to the conventional blood pressure threshold of systolic blood pressure (SBP) ≥ 140 mmHg and/or diastolic blood pressure (DBP) ≥ 90 mmHg. In several studies, participants receiving antihypertensive medication were classified as having hypertension, and some studies included physician‐diagnosed hypertension as an additional criterion. Although most of the included studies used the ≥ 140/90 mmHg threshold, a small number used alternative thresholds, including ≥ 130/80 mmHg. These differences were recorded during data extraction and were considered potential sources of clinical and methodological heterogeneity. Nevertheless, the studies were considered sufficiently comparable for synthesis because they assessed the same underlying clinical outcome, namely hypertension, using established blood pressure‐based or treatment/diagnosis‐based criteria. When alternative definitions were reported, they were considered when interpreting between‐study heterogeneity and pooled estimates.

#### 2.3.5. Study Design (D)

Observational studies with cross‐sectional, case‐control, and cohort designs were included in this review. These designs provide evidence of statistical associations between exposure and hypertension; however, particularly for the predominantly cross‐sectional evidence base, they do not establish temporality or causal relationships.

#### 2.3.6. Time Frame (T)

Studies published from database inception to January 2026 were included.

Language: Only studies published in English were included in this review.

### 2.4. Exclusion Criteria

 Population
  • Studies conducted outside Ethiopia
  • Studies involving participants < 18 years
  • Studies without a clear focus on hypertension
 Exposure
  • Studies that did not examine associations between exposures and hypertension
  • Studies where exposure–outcome relationships could not be determined
 Outcome
  • Studies where hypertension was not the primary outcome
  • Studies that did not report a measure of association (e.g., odds ratio (OR), relative risk, or equivalent)
 Study Design
  • Qualitative studies, editorials, case reports, conference abstracts, and narrative reviews
  • Experimental or modeling studies without original observational data

### 2.5. Information Sources

A thorough literature review was performed using various electronic databases to ensure the extensive coverage of relevant studies. The following databases were searched:

• PubMed/MEDLINE
• SCOPUS
• EMBASE
• Web of Science
• Cumulative Index for Nursing and Allied Health Literature (CINAHL)
• African Journals Online (SABINET)

We manually examined the reference lists of the included studies and relevant systematic reviews to identify additional articles.

### 2.6. Search Strategy

The search strategy was developed in collaboration with a seasoned information specialist. We used Medical Subject Headings (MeSH) and free‐text keywords. We consulted four key articles in tandem with our research topic and extracted their MeSH terms and free‐text keywords for analysis. We then formulated and refined our search strategy, which was pilot‐tested on PubMed. The resulting search strategy was adapted to the syntax of other databases, including MEDLINE, Scopus, Embase, Web of Science, Cumulative Index for Nursing and Allied Health Literature (CINAHL), and African Journals Online (SABINET) (Supporting File 1). The search strategy was originally developed for a broader review of East Africa’s literature. During screening, a sufficient number of eligible studies from Ethiopia were identified to justify a focused country‐level analysis. Therefore, the scope was refined to Ethiopia during the screening stage, and the original comprehensive search strategy was retained and applied to all databases.

### 2.7. Study Selection

Following the electronic database search, all identified records were exported to EndNote 21 for reference management and duplicate removal. After de‐duplication, the records were screened in two stages. First, the titles and abstracts were independently screened by two reviewers against the predefined eligibility criteria. Studies deemed irrelevant were excluded. The full texts of potentially eligible studies were retrieved and independently assessed by two reviewers for inclusion. Discrepancies between the reviewers were resolved through discussion, and when consensus could not be reached, a third reviewer was consulted. The reasons for exclusion at the full‐text stage were documented systematically. Studies that met the eligibility criteria and reported exposure–outcome associations were included. Studies were included in the quantitative meta‐analysis if sufficient homogeneity existed; otherwise, the findings were synthesized narratively. The overall study selection process is summarized in a PRISMA flow diagram (Figure 1).

**FIGURE 1:**
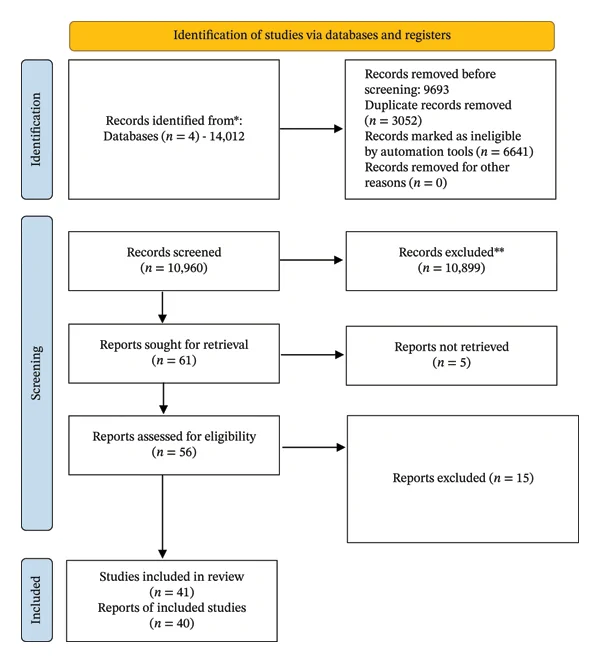
The PRISMA flow diagram.

### 2.8. Data Extraction

Data extraction was conducted using a standardized form developed for this review, including study characteristics (author, year, design, setting, and sample size), participant characteristics, exposure variables, outcome definitions, and reported measures of association. To enhance accuracy and consistency, two reviewers independently extracted data from a subset (20%) of the included studies; the remaining studies were extracted by the primary reviewer and cross‐checked for completeness. Discrepancies were resolved through discussion and arbitration by a third reviewer, if necessary. The data extraction form was iteratively refined during the process to accommodate additional relevant variables identified during the full‐text reviews. Attempts were made to contact the study authors when the data were incomplete or unclear.

### 2.9. Data Items

Using the PECODTL framework, the following data items were systematically categorized during data collection.

Study characteristics

• Author(s)
• Year of publication
• Study location (region within Ethiopia)
• Study design
• Study setting

Population characteristics

• Sample size
• Age (mean or range)
• Sex distribution

Exposure variables

Exposure variables examined in relation to hypertension were extracted and grouped as follows.

• Biological and metabolic factors (e.g., age, sex, obesity, diabetes)
• Behavioral factors (e.g., smoking, alcohol use, physical inactivity, and dietary patterns)
• Socioeconomic and psychosocial factors (e.g., education, income, occupation, stress)
• Only the exposures reported in the included studies were extracted

Outcome variable

The primary outcome was hypertension, which was defined as follows:

• SBP ≥ 140 mmHg and/or DBP ≥ 90 mmHg
• Current use of antihypertensive medication
• Physician‐diagnosed hypertension
• Variations in definitions across studies were recorded and considered a source of heterogeneity

Effect measures

Measures of association between exposures and hypertension were extracted, including

• ORs
• Risk ratios (RRs)
• Hazard ratios (HRs)
• Corresponding 95% confidence intervals (CIs). Where available, the adjusted estimates were prioritized.

Additional methodological variables

• Statistical models used (e.g., univariable or multivariable)
• Sampling approach
• Duration of follow‐up (for longitudinal studies)

### 2.10. Exposure Definition and Classification

Exposure variables were defined as demographic, behavioral, metabolic, and socioeconomic characteristics examined in relation to the risk of hypertension. Variables were extracted as reported and grouped into broader domains, namely metabolic, behavioral, and socioeconomic/psychosocial, to support a structured synthesis [13]. A harmonization process was applied to address the variability in exposure categorization and reference groups. Where necessary, exposures were standardized into consistent contrasts, and reference groups were aligned to ensure a uniform direction of effect, following the principles of the Global Burden of Disease comparative risk assessment [19]. Transformations were conducted at the study level using reported estimates with documented assumptions. Variations in exposure definitions were retained and considered sources of heterogeneity.

### 2.11. Risk of Bias Assessment

The methodological quality was assessed using the JBI critical appraisal checklists for analytical cross‐sectional and case–control studies [24]. The studies were evaluated across key domains, including participant selection, measurement of exposure and outcomes, control of confounding factors, and reporting clarity. Selection bias was assessed based on representativeness and recruitment, information bias regarding the validity and reliability of the measurements, and confounding in the identification and adjustment of relevant variables. Reporting bias was assessed based on the completeness and transparency of the results. Quality assessments were used to evaluate the interpretation and overall confidence in the evidence.

### 2.12. Summary Measures

Associations between exposure and hypertension were summarized using reported effect estimates, primarily ORs, with adjusted estimates prioritized where available. Corresponding 95% CIs were extracted to reflect the precision. The estimates were interpreted based on the direction, magnitude, and consistency across studies without predefined thresholds. ORs > 1.0 indicated a positive association, whereas ORs < 1.0 indicated an inverse (protective) association. Interpretation was performed cautiously, considering the study design, methodological limitations, and between‐study heterogeneity, in line with the established epidemiological guidance [25].

### 2.13. Data Synthesis and Analysis

Data synthesis combined narrative and quantitative approaches to the analysis. Study characteristics were summarized descriptively using frequencies and percentages, and the findings were presented in structured evidence tables. Where sufficient comparability existed, random‐effects meta‐analyses using inverse‐variance weighting were performed to estimate pooled associations between exposure and hypertension using ORs [13]. Random‐effects meta‐analysis was used for the primary pooled estimates because the included studies were expected to differ in population characteristics, study settings, exposure measurement, outcome ascertainment, and analytical approaches, such that a common underlying effect could not be reasonably assumed. Between‐study variance (*τ* ^2^) was estimated using restricted maximum likelihood (REML), and Hartung–Knapp adjustment was applied to provide a more conservative inference, particularly where the number of contributing studies was limited or heterogeneity was substantial. Therefore, the random‐effects model accounts for both within‐study sampling errors and between‐study variability.

Pooling was based on a common underlying outcome definition rather than requiring identical operational criteria across all studies. Because most studies used the same ≥ 140/90 mmHg threshold, and the remaining definitions represented established clinical approaches to identifying hypertension, the variation was judged unlikely to preclude quantitative synthesis. Nevertheless, definition‐related variations were treated as potential contributors to heterogeneity and were considered when interpreting the pooled associations. Statistical heterogeneity was quantified using the *I* ^2^ statistic. Given the expected clinical and methodological diversity across studies, heterogeneity was interpreted in light of differences in study populations, geographical settings, study periods, hypertension ascertainment, exposure definitions and measurements, and analytical approaches. However, a formal investigation of these potential sources using meta‐regression or systematic subgroup analyses was not undertaken because the number of studies varied substantially across individual factor‐specific meta‐analyses, with several factors represented by relatively few studies. Accordingly, *I* ^2^ was used to characterize statistical heterogeneity, while potential clinical and methodological contributors were considered qualitatively rather than treated as empirically established explanations of between‐study variations.

To address the variability in exposure categorization and reference groups, effect estimates were harmonized to ensure comparability. Where necessary, ORs were transformed to maintain a consistent direction of association, with all estimates reflecting increased odds of hypertension relative to a common reference group, following established approaches in comparative risk assessments [19]. Leave‐one‐out sensitivity analyses were conducted to examine the influence of individual studies on pooled estimates, and Baujat plots were used as influence diagnostics, where applicable [26]. Potential small‐study effects were assessed using funnel plots and Egger’s regression test, where the number of contributing studies was considered sufficient for assessment [26]. Given the limited statistical power of funnel plot asymmetry tests when relatively few studies are available, these methods were interpreted as assessments of potential small‐study effects rather than definitive tests of publication bias. The certainty of evidence was assessed only for factors associated with hypertension in the meta‐analysis. Coincidentally, at least 10 studies were involved in the meta‐analysis of each of these factors, and as a result, Egger’s test was interpreted as a reliable indicator of publication bias.

In addition, a quantitative framework based on association weight estimates was applied to support the comparative interpretation. Association weights were derived from the product of pooled effect estimates (ORs) and a consistency measure that reflected the proportion of studies reporting statistically significant associations [27]. These were used to compare the relative strength and consistency of associations across factors, with grouping based on the percentile distributions. Cumulative association‐weight distributions were further examined to identify the levels at which combinations of factors showed relatively high cumulative association values. These thresholds were defined empirically and used as interpretive tools to explore patterns of co‐occurrence among factors relevant to population‐level prevention strategies [28, 29]. All framework‐based analyses are exploratory and should be interpreted cautiously, given the observational nature of the underlying evidence and predominance of cross‐sectional studies. The computation of association weight, association responsiveness, empirical association thresholds, interaction density, and geotemporal trends (GTT) followed previously described methods developed in prior meta‐analytic modeling studies [27, 30, 31]. Analyses were conducted using R (version 4.4.5), with statistical significance set at *α* = 0.05.

### 2.14. Grading the Strength of Evidence

The certainty of the evidence was assessed using the GRADE framework [32] across five domains: risk of bias, inconsistency, indirectness, imprecision, and publication bias. The risk of bias was evaluated using the JBI critical appraisal tool [24]. Inconsistency was assessed by variability in effect estimates (CI overlap and *I* ^2^), indirectness by alignment with the review question, and imprecision by sample size, event counts, and CI width, particularly when the estimates crossed zero. Publication bias was examined using funnel plots and Egger’s test. Overall, the certainty was rated as high, moderate, low, or very low, in accordance with the GRADE guidance for observational studies [31].

## 3. Results

### 3.1. Study Selection

A total of 14,012 records were identified through database searches, of which 3052 were duplicates. The remaining 10,960 records were screened by title and abstract, resulting in the exclusion of 10,899 records that did not meet eligibility criteria. Full‐text screening was conducted for 56 articles, of which 41 met the inclusion criteria and were included in this review (Figure 1).

The included studies examined a wide range of factors associated with hypertension, which were grouped into demographic, socioeconomic, behavioral, and metabolic domains. Demographic variables included age (36 studies), sex (30 studies), family history of hypertension (18 studies), geographic residence (6 studies), and ethnicity (1 study). Socioeconomic factors included education (18), occupation (9), income (9), wealth status (5), and marital status (18). Behavioral factors included alcohol consumption (28 studies), physical inactivity (25 studies), tobacco use (4 studies), khat chewing (10 studies), prolonged sitting (3 studies), transport‐related activity (1 study), and sleep duration (2 studies). The dietary variables included salt intake (10), overall dietary patterns (8), fruit and vegetable intake (12), cooking oil type (4), and meat consumption (2). Metabolic and anthropometric factors were the most frequently reported, including body mass index (BMI) (37 studies), obesity (8 studies), waist–hip ratio (6 studies), diabetes (16 studies), blood glucose (2 studies), and lipid profiles (4 studies). A small number of studies have assessed clinical or treatment‐related factors such as antiretroviral therapy (1) (Table 1).

**TABLE 1:** Study characteristics.

Author & year	Definition of HTN	Age (yr)	Female sex (%)	Race/ethnicity	Design	Sample size	Settings	Region	Country
Essa et al. 2022 [33]	SBP ≥ 140 and/or DBP ≥ 90 mmHg or on medication	36.32 ± 12.48	55	Debre Markos town	cross‐ sectional study	630	Community‐based	North‐West	Ethiopia
Dereje et al. 2020 [34]	SBP ≥ 140 and/or DBP ≥ 90 mmHg or on medication	36 ± 11.6	41.5	Hosanna town	Cross‐sectional	634	Community‐based	Southern	Ethiopia
Endrias et al. 2024 [35]	SBP ≥ 140 and/or DBP ≥ 90 mmHg or on medication	NR	23.8	Wolaita zone	Cross sectional	417	Hospital‐based	Southern	Ethiopia
Gebremedin et al. 2021 [36]	SBP ≥ 140 and/or DBP ≥ 90 mmHg or on medication	55 years	47.5	Jimma, Southwest	Unmatched case control	406	University medical center	South‐West	Ethiopia
Gebremeskel et al. 2024 [37]	SBP ≥ 140 and/or DBP ≥ 90 mmHg or on medication	NR	38.3	Tigrai Regional state	Cross sectional	240	Aksum University	Northern	Ethiopia
Haile and Geset Haile 2021 [38]	Raised BP per WHO STEPS	36.4 ± 9.1	51.6	Debre Berhan town	Cross sectional	680	Community	Amhara North‐West	Ethiopia
Solela et al. 2024 [39]	SBP ≥ 140 and/or DBP ≥ 90 mmHg	47.6 ± 13.5	37.5	Yekatit	Cross sectional	301	Hospital medical college	Addis Ababa	Ethiopia
Zekewos et al. 2019 [40]	SBP ≥ 140 and/or DBP ≥ 90 mmHg or on medication	40 (30–54)	46.6	Bona district	Cross‐sectional	1952	Community	Southern	Ethiopia
Abebe et al. 2015 [41]	SBP ≥ 140 and/or DBP ≥ 90 mmHg	47 ± 12.4		Northwest Ethiopia (community)	Cross‐sectional	2200	Community‐based	Amhara North‐West	Ethiopia
Abebe and Yallew 2019 [42]	≥ 140/90 mmHg	—	46.2	Addis Ababa (adult outpatient clients)	Cross‐sectional	487	Facility‐based (outpatient)	Addis Ababa	Ethiopia
Adal et al. 2024 [43]	≥ 140/90 mmHg	—	0	Addis Ababa terminals (long‐distance bus drivers)	Cross‐sectional	391	Workplace/terminal‐based	Addis Ababa	Ethiopia
Anjulo et al. 2021 [44]	≥ 140/90 mmHg	—		Areka town, Wolaita Zone	Cross‐sectional	581	Community‐based	Southern	Ethiopia
Asemu et al. 2021 [45]	JNC VII; mean of 2nd & 3rd BP readings; typically ≥ 140/90 mmHg	32 (median; IQR 25–45)	57.3	Addis Ababa (community)	Cross‐sectional	3560	Community‐based	Addis Ababa	Ethiopia
Asfaw et al. 2018 [46]	≥ 140/90 mmHg			Hosanna town, Hadiya Zone	Cross‐sectional	525	Community‐based	Southern	Ethiopia
Asresahegn et al. 2017 [47]	SBP ≥ 140 or DBP ≥ 90 or meds	25–65	NR	NR	Cross‐sectional	492	Community‐based	Somali Eastern Ethiopia	Ethiopia
Awoke et al. 2012 [48]	SBP ≥ 140 or DBP ≥ 90 or meds	≥ 35	NR	NR	Cross‐sectional	679	Community‐based	Amhara North‐West	Ethiopia
Ayalew and Wale 2022 [49]	SBP ≥ 130 or DBP ≥ 80	≥ 18	NR	NR	Cross‐sectional	644	Community‐based	Wolaita zone Southern Ethiopia	Ethiopia
Badego et al. 2020 [50]	≥ 140/90 mmHg			Cross sectional	546	Institution‐based	Sidama zone	Ethiopia	
Bayray et al. 2018 [51]	SBP ≥ 140 or DBP ≥ 90	Adults	NR	NR	Cross‐sectional	1525	Institution‐based	Tigray Region, Nothern Ethiopia	Ethiopia
Belachew et al. 2018 [52]	SBP ≥ 140 or DBP ≥ 90	≥ 18	NR	NR	Cross‐sectional	308	Hospital‐based	Amhara North‐West	Ethiopia
Belay et al. 2022 [53]	SBP ≥ 140 or DBP ≥ 90	Adults	NR	NR	Cross‐sectional	449	Hospital‐based	North‐West	Ethiopia
Berhe et al. 2020 [54]	SBP ≥ 140 or DBP ≥ 90	≥ 25	NR	NR	Cross‐sectional	414	Hospital‐based	Addis Ababa	Ethiopia
Birhan et al. 2022 [31]	Clinical diagnosis	Adults	NR	NR	Case–control	128	Hospital‐based	Amhara North‐West	Ethiopia
Bonsa et al. 2014 [55]	SBP ≥ 140 or DBP ≥ 90 or meds	≥ 15	NR	NR	Cross‐sectional	396	Community‐based	Oromia South‐West	Ethiopia
Dejenie et al. 2021 [56]	SBP ≥ 130 or DBP ≥ 80	≥ 18	NR	NR	Cross‐sectional	513	Institution‐based	Amhara North‐West	Ethiopia
Feyisa et al. 2023 [57]	SBP ≥ 140 or DBP ≥ 90	Adults	NR	NR	Cross‐sectional	578	Institution‐based	Oromia South‐West	Ethiopia
Gedamu and Sisay 2021 [58]	SBP ≥ 140 or DBP ≥ 90	≥ 18	NR	NR	Cross‐sectional	627	Institution‐based	Amhara North‐West	Ethiopia
Gedefaw et al. 2025 [59]	PH defined by echocardiography	RHD patients	NR	NR	Prospective cross‐sectional	310	Hospital‐based	Amhara North‐West	Ethiopia
Gelassa et al. 2022 [60]	SBP ≥ 140 or DBP ≥ 90	Adults	NR	NR	Cross‐sectional	605	Community‐based	Central Ethiopia	Ethiopia
Geleta et al. 2019 [61]	SBP ≥ 140 or DBP ≥ 90	≥ 18	NR	NR	Cross‐sectional	711	Community‐based	Oromia South‐West	Ethiopia
Gudina et al. 2013 [62]	SBP ≥ 140 or DBP ≥ 90	≥ 15	NR	NR	Cross‐sectional	734	Hospital‐based	Oromia South‐West	Ethiopia
Gutema et al. 2020 [63]	SBP ≥ 135 or DBP ≥ 85	25–64	NR	NR	Cross‐sectional	3368	Community‐based	Southern	Ethiopia
Haile et al. 2024 [62, 64]	SBP ≥ 140 or DBP ≥ 90	≥ 18 (PLHIV)	NR	NR	Cross‐sectional	411	Hospital‐based	Addis Ababa	Ethiopia
Helelo et al. 2014 [65]	SBP ≥ 140 or DBP ≥ 90 or on medication	≥ 18	NR	NR	Community‐based cross‐sectional	734	Community‐based	Southern	Ethiopia
Kassie et al. 2020 [66]	SBP ≥ 140 or DBP ≥ 90 or on medication	≥ 18	NR	NR	Community‐based cross‐sectional	804	Community‐based	Amhara North‐West	Ethiopia
Kiber et al. 2019 [67]	SBP ≥ 140 or DBP ≥ 90 or on medication	≥ 18	NR	NR	Community‐based cross‐sectional	627	Community‐based	Amhara North‐West	Ethiopia
Kumma et al. 2021 [68]	SBP ≥ 140 or DBP ≥ 90 or on medication	≥ 18	NR	NR	Community‐based cross‐sectional	610	Community‐based	Southern	Ethiopia
Paulose et al. 2022 [69]	SBP ≥ 140 or DBP ≥ 90 or on medication	≥ 18	NR	NR	Community‐based cross‐sectional	612	Community‐based	Addis Ababa	Ethiopia
Rike et al. 2022 [70]	SBP ≥ 140 or DBP ≥ 90	≥ 18	0	NR	Institution‐based cross‐sectional	415	Institution‐based	Oromia South‐West	Ethiopia
Saif‐Ur‐Rahman et al. 2021 [71]	SBP ≥ 140 or DBP ≥ 90 or on medication	25–64	40.4	NR	Institution‐based cross‐sectional	1380	Institution‐based	Amhara North‐West	Ethiopia
Tadesse and Alemu 2014 [72]	SBP ≥ 140 or DBP ≥ 90	≥ 18	25.6	NR	Institution‐based cross‐sectional	610	Institution‐based	Amhara North‐West	Ethiopia

Abbreviation: nr, not reported.

### 3.2. Study Characteristics

Forty‐one studies were included in this review, comprising two case‐control studies [38, 39] and 39 observational studies, predominantly cross‐sectional designs. The studies were conducted in adult populations across multiple regions of Ethiopia and included both community‐ and institution‐based samples with diverse participant characteristics. Geographically, the included studies provided broad coverage across different regions of the country, although the number of studies varied according to location. Most studies were community‐based and utilized structured questionnaires alongside blood pressure measurements, whereas a smaller proportion were hospital‐based or case‐control studies. Overall, the included studies demonstrated variability in study design, population characteristics, exposure definitions, and analytical approaches, reflecting the heterogeneity of the available evidence. A detailed summary of the study characteristics is presented in Table 1.

### 3.3. Definition of Hypertension

The operational definition of hypertension was consistent across the included studies. Most (37/41) studies used SBP ≥ 140 mmHg and/or DBP ≥ 90 mmHg, with several also including current antihypertensive medication use. A small number of studies used physician‐diagnosed hypertension or alternative blood pressure thresholds, including ≥ 130/80 mmHg. These variations were considered potential sources of clinical heterogeneity but did not preclude synthesis because the predominant outcome definitions were consistent across studies. The detailed definitions of each study are presented in Table 1.

### 3.4. Risk of Bias

Most of the included studies were assessed as having low to moderate methodological limitations based on the appraisal criteria applied. Many studies met the key quality domains, including clear inclusion criteria, appropriate measurement of exposures and outcomes, and use of statistical methods to account for potential confounding. However, some studies have demonstrated limitations, particularly in the identification and control of confounding variables, clarity of exposure measurement, and reporting of analytical approaches. Overall, the methodological quality of the included studies was considered acceptable for synthesis, although variability in the study design and measurement approaches was noted. Therefore, the findings should be interpreted in the context of these limitations of the study. A detailed summary of the risk of bias assessment is provided in Supporting Table 2.

### 3.5. Risk Factor Association With HTN (Narrative Synthesis)

#### 3.5.1. Demographic and Contextual Factors

##### 3.5.1.1. Age and Sex

Eighteen studies assessed age and consistently showed higher odds of hypertension with increasing age. Large effect sizes were reported in the categorical analyses (e.g., ≥ 50 years), whereas the continuous models showed smaller incremental increases, indicating a dose–response pattern. Despite the variability in magnitude, the direction of the association was consistently positive (Supporting File 3). Fifteen studies examined sex, and the findings showed substantial heterogeneity. In addition, several studies have reported higher odds among men, whereas others have shown lower or nonsignificant associations. Overall, no consistent independent association was observed between sex and the risk of hypertension (Supporting File 3).

##### 3.5.1.2. Ethnicity and Residence

Evidence was limited to a single study that reported variations in ethnic groups. Interpretation was constrained by imprecision and limited data (Supporting File 3). Six studies assessed residence, with largely nonsignificant and inconsistent findings. Overall, residence was not consistently associated with hypertension (Supporting Table 3).

##### 3.5.1.3. Family History of Hypertension and Diabetes

Eighteen studies assessed the family history of hypertension and reported higher odds of hypertension among individuals with a positive family history. Although the effect sizes varied, the direction of the association was consistently positive across most studies (Supporting File 3). Two studies examined the family history of diabetes and suggested higher odds of hypertension among individuals with a positive family history, although the evidence remains limited (Supporting File 3).

#### 3.5.2. Socioeconomic and Psychosocial Factors

##### 3.5.2.1. Education and Occupation/Employment Status

Eighteen studies assessed education, and the findings showed heterogeneity. Lower educational attainment was generally associated with higher odds of hypertension, although several studies have reported weak or nonsignificant associations. Overall, the direction of the association was predominantly positive but inconsistent (Supporting File 3). Nine studies assessed occupation and reported heterogeneous findings. Higher odds of hypertension were reported among unemployed, retired, or manual workers in some studies, while others reported no significant associations. Overall, the association was positive but inconsistent (Supporting File 3).

##### 3.5.2.2. Income and Wealth Index

Nine studies examined income, with mixed findings. Lower income was associated with higher odds of hypertension in several studies, whereas others have reported weak or nonsignificant associations. Overall, the evidence suggests a positive but inconsistent association (Supporting Table 3). The wealth index was not consistently assessed, and socioeconomic position was primarily measured using income categories only. Therefore, evidence on wealth and hypertension is limited, and the findings should be interpreted cautiously (Supporting File 3).

##### 3.5.2.3. Marital Status and Family/Household Size

Twelve studies examined marital status and reported mixed findings. Some studies have reported higher odds among married or previously married individuals, whereas others have found no significant associations. Overall, the evidence suggests a positive but inconsistent relationship (Supporting Table 3). Three studies assessed household size, with inconsistent results. One study reported higher odds in larger households, whereas others found weak or nonsignificant associations. However, the evidence remains limited (Supporting File 3).

##### 3.5.2.4. Knowledge and Awareness of Hypertension

Six studies assessed knowledge, with variable findings. Poor knowledge was associated with higher odds of hypertension in several studies, although this was not consistently observed. Overall, the association was positive but inconsistent (Supporting File 3).

#### 3.5.3. Behavioral and Lifestyle Factors

##### 3.5.3.1. Alcohol Use and Smoking

Twenty‐eight studies assessed alcohol use, and their findings showed substantial heterogeneity in the results. Several studies have reported higher odds of hypertension among alcohol users, whereas others have found weak, nonsignificant, or inverse associations. Overall, alcohol use showed a positive but inconsistent association (Supporting File 3). Fifteen studies examined smoking, with mixed findings. Some studies reported higher odds among smokers, whereas others showed weak, inverse, or nonsignificant associations. Overall, the evidence indicates an inconsistent relationship (Supporting File 3).

##### 3.5.3.2. Tobacco Use (Noncigarette or Combined) and Khat Chewing

Four studies assessed broader tobacco use and consistently reported higher odds of hypertension in tobacco users. Although the number of studies was limited, the direction of the association was consistently positive (Supporting File 3). Ten studies assessed khat chewing and reported heterogeneous findings. While several studies reported higher odds among users, others have shown inverse or nonsignificant associations. Overall, the evidence suggests a mixed and inconsistent relationship (Supporting Table 3).

##### 3.5.3.3. Physical Activity and Sedentary Behavior (Time Spent Sitting) and Mode of Transport

Twenty‐seven studies examined physical activity, and their findings were heterogeneous. Lower activity levels were associated with higher odds of hypertension in several studies, whereas others have reported protective or nonsignificant associations. Overall, this relationship was inconsistent across studies (Supporting File 3). Evidence from a single study suggested a modest, nonsignificant increase in hypertension among individuals with prolonged sitting times. One study assessed transport modes and reported higher odds of hypertension among individuals using motorized transport than among those using active transportation. However, the evidence remains limited (Supporting File 3).

##### 3.5.3.4. Sleep Duration

Two studies examined sleep duration and consistently reported higher odds of hypertension among individuals with short sleep duration. Despite the limited evidence, the direction of the association was consistent (Supporting File 3).

#### 3.5.4. Dietary Factors

##### 3.5.4.1. Salt Intake

Ten studies examined salt intake, and generally higher odds of hypertension were reported among individuals with higher intake. However, some studies have reported weak or nonsignificant associations between these variables. Overall, the evidence suggests a predominantly positive but heterogeneous association (Supporting Table 3).

##### 3.5.4.2. Fruit and Vegetable Intake

Twelve studies assessed fruit and vegetable consumption, with heterogeneous results. Lower intake was associated with a higher odd of hypertension in several studies, whereas others reported protective or nonsignificant associations. Overall, this relationship was inconsistent (Supporting Table 3).

##### 3.5.4.3. Meat Consumption

Two studies examined meat consumption with mixed results. One study reported higher odds of fatty meat intake, whereas another study showed no clear association. However, the evidence remains limited and heterogeneous (Supporting File 3).

##### 3.5.4.4. Type of Cooking Oil

Four studies assessed cooking oil type, with findings suggesting higher odds of hypertension associated with less healthy oil types in some studies, while others reported modest or imprecise associations. Overall, the evidence was limited and inconsistent (Supporting File 3).

#### 3.5.5. Metabolic and Clinical Biomarkers

##### 3.5.5.1. BMI and Obesity

Six estimates assessed BMI as a continuous variable, with higher odds of hypertension observed at higher BMI levels. Despite variations in magnitude, the direction of the association was predominantly positive (Supporting Table 3). Fourteen studies examined obesity, most of which reported higher odds of hypertension in obese individuals than in nonobese individuals. Although the effect sizes varied, the association was consistently positive (Supporting Table 3).

##### 3.5.5.2. Waist Circumference and Waist‐to‐Height Ratio

Nine studies assessed waist circumference, and higher odds of hypertension were reported among individuals with central obesity in several studies, although some reported weak or nonsignificant findings. Overall, the association was positive but heterogeneous (Supporting File 3). One study examined the waist‐to‐height ratio and reported higher odds of hypertension among individuals with elevated ratios. The evidence is limited but suggests a positive association between these factors (Supporting File 3).

##### 3.5.5.3. Raised Blood Glucose and Diabetes Mellitus

Two studies assessed blood glucose as a continuous measure and reported higher odds of hypertension with increasing levels. The evidence was limited but consistent (Supporting File 3). Sixteen studies assessed diabetes, most of which reported higher odds of hypertension in individuals with diabetes. Although the effect sizes varied widely, this association was consistently positive (Supporting File 3).

##### 3.5.5.4. Triglycerides and Cholesterol

Four studies each assessed triglyceride and cholesterol levels, with generally higher odds of hypertension reported for elevated levels, although the findings were heterogeneous. Overall, the association was positive but varied (Supporting File 3).

##### 3.5.5.5. Antiretroviral Therapy (ART) and Contraceptive Use

One study assessed ART exposure and reported higher odds of hypertension with a longer duration of treatment, whereas the regimen‐specific findings were mixed. However, the evidence is limited (Supporting File 3). Two studies have examined contraceptive use, with conflicting findings. One study reported lower odds, whereas another reported substantially higher odds. Overall, the evidence is limited and inconsistent (Supporting File 3).

##### 3.5.5.6. Other Reported Factors

Eleven studies examined diverse additional factors with highly variable findings. Some exposures showed strong positive associations, whereas others were protective or nonsignificant. Overall, this category reflected heterogeneous and context‐specific associations (Supporting File 3), including:

#### 3.5.6. Meta‐Analysis of Factors Associated With Hypertension

Across the analyzed exposures, most factors demonstrated statistically significant associations with hypertension, although heterogeneity was consistently high. The results are summarized as follows.

##### 3.5.6.1. Demographic Factors

Older age (≥ 50 years) was associated with increased odds of hypertension (OR 3.84, 95% CI 2.22–6.65; *I* ^2^ = 88.6%). Sex was not significantly associated with hypertension (OR 1.06, 95% CI 0.85–1.31), with substantial heterogeneity.

##### 3.5.6.2. Behavioral Factors

Alcohol use (OR 1.72, 95% CI 1.32–2.23), smoking (OR 1.28, 95% CI 0.85–1.93), tobacco use (OR 3.81, 95% CI 1.69–8.58), khat chewing (OR 1.75, 95% CI 0.83–371, imprecise), physical inactivity (OR 1.41, 95% CI 0.77–2.63), time spent sitting (OR 1.10, 95% CI 0.14–8.65), and abnormal sleep duration (OR 2.38, 95% CI 0.05–105.45) were associated with higher odds of hypertension. However, several estimates, particularly those for khat chewing, sitting time, and sleep duration, showed wide CIs, indicating substantial imprecision. Heterogeneity was high across the analyses.

##### 3.5.6.3. Dietary Factors

High salt intake (OR, 1.95; 95% CI, 0.92–4.12), poor dietary patterns (OR, 0.54; 95% CI, 0.24–1.20), low fruit and vegetable intake (OR, 0.67; 95% CI, 0.51–0.89), and fatty meat consumption (OR, 1.45; 95% CI, 0.01–208.69) were associated with hypertension. While some associations suggested an increased risk, others indicated protective or nonsignificant effects of the drugs. Several estimates, particularly those for meat consumption, were highly imprecise and should be interpreted cautiously.

##### 3.5.6.4. Socioeconomic Factors

Lower educational attainment was not significantly associated with hypertension (OR 0.98, 95% CI 0.72–1.34), despite substantial heterogeneity (*I* ^2^ = 93.0%). Marital status (OR 1.58, 95% CI 1.21–2.07) was associated with increased odds of hypertension, whereas residence (urban vs. rural) showed no significant association (OR 0.94, 95% CI 0.57–1.54). Poor knowledge of hypertension (OR 1.48, 95% CI 0.28–7.71) and family size (OR 1.15, 95% CI 0.42–3.18) showed nonsignificant and imprecise associations.

##### 3.5.6.5. Metabolic Factors

Obesity (OR 2.07, 95% CI 1.47–2.93), increased cholesterol (OR 1.86, 95% CI 1.48–2.32), and diabetes (OR 4.08, 95% CI 2.91–5.72) were associated with higher odds of hypertension.

BMI categories showed differential associations, with overweight being associated with increased risk (OR 2.08, 95% CI 1.47–2.94), while being underweight was associated with lower odds (OR 0.51, 95% CI: 0.27–0.98). Waist circumference showed no clear association (OR 1.00, 95% CI 0.47–2.12), while waist–hip ratio suggested an increased risk (OR 2.52, 95% CI 0.23–27.07); however, these estimates were imprecise.

##### 3.5.6.6. Familial and Clinical Factors

A family history of hypertension was associated with increased odds of hypertension (OR 2.91, 95% CI 1.91–4.42). A family history of diabetes (OR 2.74, 95% CI 0.00–19451.95) and contraceptive use (OR 4.32, 95% CI 0.00–158935.0) showed highly imprecise estimates with extremely wide CIs, limiting their interpretability.

##### 3.5.6.7. Publication Bias and Sensitivity Analysis

For most exposures with sufficient studies, Egger’s test did not indicate strong evidence of small study effects. Exceptions included education (*p* < 0.001) and family history of hypertension (*p* = 0.014), in which potential asymmetry was observed. Sensitivity analyses using leave‐one‐out methods showed that the pooled estimates were generally robust, with no single study exerting a dominant effect.

#### 3.5.7. Risk Responsiveness and Geotemporal Pattern

Risk responsiveness (Ri) and GTT were used as exploratory metrics to assess the consistency and distribution of the evidence across studies. Several factors demonstrated high or near‐complete responsiveness, indicating consistent statistical associations across the contributing studies. These included age, tobacco use, sleep duration, waist–hip ratio, and other selected clinical factors. Other factors, including diabetes, education, alcohol use, dietary patterns, and adiposity measures, showed moderate responsiveness, reflecting consistent but variable associations. Lower responsiveness was observed for smoking, salt intake, sex, and family size, indicating less stable or inconsistent results. Geotemporal patterns showed that some factors, including age, diabetes, alcohol use, and tobacco use, were reported across multiple settings and time periods, suggesting a broader evidence coverage. In contrast, other factors such as residence, meat consumption, and family size demonstrated limited geographic and temporal representation. Overall, these patterns indicate variability in both the consistency and distribution of evidence across risk factors, with some factors showing stronger and more widely represented associations than others (Table 2).

**TABLE 2:** Meta‐analysis of risk factors of associated with hypertension in Ethiopia.

Factors	Reference	Pooled odds ratio	95% CI	*I* ^2^ (%)	Risk responsiveness (Ri)	Risk weight (Rw)	Risk class	Crw	Geo‐coverage	Temporal coverage	Geo‐temporal trend (GTT)
Age (≥ 50)	< 50	3.84	(2.22–6.65)	88.6	1.00	3.84	First class	3.84	0.36	0.67	0.620
Alcohol	No alcohol use	1.72	1.32–2.23	85.2	0.781	1.343	Second class	5.183	0.5	0.67	0.557
BMI (underweight)	Normal weight	0.51	0.27–0.98	79.4	0.588	0.3	Third class	5.483	0.571	0.67	0.485
BMI (overweight)	Normal weight	2.08	1.47–2.94	91.4	0.774	0.372	Third class	6.227	0.588	0.67	0.583
Cholesterol	No raised cholesterol	1.86	1.48–2.32	54.4	0.75	1.395	Second class	7.622	0.286	0.67	0.469
Contraceptive use	No	4.32	0.00–158935.0	92.7	1.00	—	—	7.622	0.143	0.67	0.548
Diabetes	No diabetes	4.08	2.91–5.72	63.5%	0.875	3.57	First class	11.192	0.5	0.67	0.604
Dietary habit	Good diet	0.54	0.24–1.20	92.6	0.75	—	—	11.192	0.429	0.67	0.893
Education No formal education	Formal education	0.98	0.72–1.34	93.0	0.786	0.77	Second class	11.962		0.67	0.250
Family history of diabetes	No family History of diabetes	2.74	0.00–19451.95	91.81	1.000	—	—	11.962	0.143	0.33	0.524
Family history of hypertension	No family history of hypertension	2.91	1.91–4.42	84.1	0.889	2.59	Second class	14.552	0.429	0.67	0.588
Family size	Small family size	1.15	0.42–3.18	74.6	0.333	—	—	14.552	0.214	0.67	0.238
Fruits and veggies	Sufficient intake	0.67	0.51–0.89	58.3	0.583	—	—	14.552	0.333	0.67	0.402
Gender (male)	Female	1.06	0.85–1.31	75.3	0.333	—	—	14.552	0.5	0.67	0.333
Khat chewing (yes)	No khat chewing	1.75	0.83–3.71	89.8	0.6	—	—	14.552	0.5	067	0.67
Knowledge of hypertension (poor)	Good knowledge	1.48	0.28–7.71	97.4	0.833	—	—	14.552	0.33	0.67	0.528
Marital status	Never married/single	1.58	1.21–2.07	64.1	0.474	0.748	Third class	15.30	0.429	0.67	0.933
Meat consumption (yes fatty meat)	No	1.45	0.01–208.69	30.3	0.500	—	—	15.30	0.071	0.33	0.262
Obesity	Nonobese	2.07	1.47–2.93	66.1	0.625	1.293	Third class	16.593	0.429	0.67	0.455
Physically inactivity	Physically active	1.41	0.77–2.63	90.9	0.625	—	—	16.593	0.357	0.67	0.432
Residence (urban)	Rural	0.94	0.57–1.54	61.9	—	—	—	16.593	0.214	0.67	0.071
Salt intake (yes/high)	No (low, optimal)	1.95	0.92–4.12	86.9	0.444	—	—	17.037	0.429	0.67	0.365
Sleep duration	Adequate/normal sleep	2.38	0.05–105.45	50.2	1.000	—	—	17.037	0.143	0.67	0.548
Smoking (yes)	No smoking	1.28	0.85–1.93	78.6	0.467	—	—	17.037	0.429	0.67	0.783
Time spent sitting	Low sitting time	1.10	0.14–8.65	91.8	0.667	—	—	17.037	0.143	0.33	0.357
Tobacco use (yes)	No tobacco	3.81	1.69–8.58	52.6	1.00	3.81	First class	20.847	0.286	0.67	0.595
Waist circumference	Normal waist circumference	1.00	0.47–2.12	98.3	0.625	—	—	20.847	0.286	0.67	0.408
Waist–hip ratio	Normal WHR	2.52	0.23–27.07	96.5	1.00	—	—	20.847	0.214	0.67	0.571

#### 3.5.8. Risk Stratification and Critical Risk Thresholds

Cumulative risk weight (CRW) distributions were examined to explore the patterns of combined risk across the factors. Percentile‐based thresholds were used to describe the relative cumulative risk levels within the dataset. The 50th and 75th percentiles were used to define the lower, intermediate, and higher cumulative‐risk groups. Most CRW values were concentrated within the lower‐risk range, with fewer observations in higher‐risk categories. These distributions provide a descriptive overview of how risk factors cluster within the study population. These analyses were exploratory and intended to support the comparative interpretation of risk patterns rather than define clinical thresholds or guide individual‐level decision‐making (Table 2; Figure 2).

**FIGURE 2:**
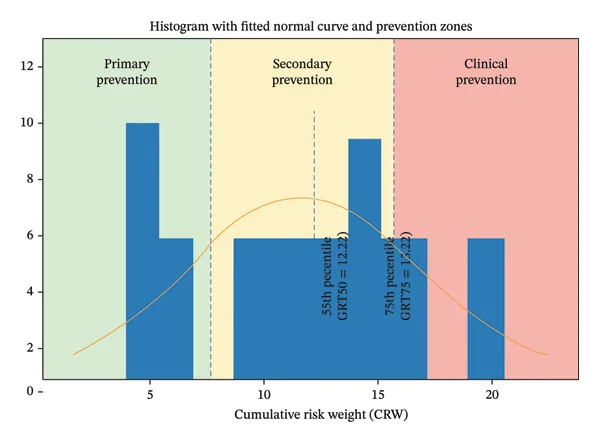
Histogram showing zones of prevention.

#### 3.5.9. Interaction Patterns and Parsimonious Combinations of Hypertension Risk Factors

Within the analytical framework, the risk of hypertension was examined as a cumulative process in which combinations of risk factors contributed to increasing levels of overall risk relative to empirically defined thresholds. This approach allowed for the exploration of interaction patterns and parsimony across combinations of factors. In the first set of parsimonious combinations, relatively small sets of four to five factors were sufficient to exceed the median critical risk threshold (CRT_50_ = 12.21). These findings suggest that the risk of hypertension may be represented by compact combinations of factors rather than requiring extensive multivariate structures.

Certain factors, particularly older age (≥ 50 years), diabetes, and tobacco use, appeared consistently across these combinations. Other factors, including adiposity measures, cholesterol, alcohol use, and family history, were variably present across combinations, contributing to additional risk in different configurations. Social variables, such as education and marital status, were also observed within some combinations, contributing to the variability in risk profiles without substantially increasing the number of interacting factors. Behavioral factors, including tobacco and alcohol use, showed variable inclusion across combinations, although tobacco use appeared more frequently in high‐risk groupings.

Overall, the observed patterns indicate that hypertension risk may be structured around a limited number of recurring factor combinations, with consistent involvement of demographic and metabolic factors and more variable contributions from behavioral and social factors. These findings provide an exploratory representation of how risk factors may cluster and interact within the study context (Table 3; Figure 3).

**TABLE 3:** Hypertension prediction from risk factor and checklist combination.

Rothman’s causal paths	Risk‐factor combination	ΣRw	*k*	ΣGTT	Interaction count	Interaction density	Interaction pairs
M1	Age ⟶ Diabetes ⟶ Tobacco (+Khat)	12.27	4	2.489	6	0.133	Age–Diabetes; Age–Tobacco; Age–Khat chewing; Diabetes–Tobacco; Diabetes–Khat chewing; Tobacco–Khat chewing
M2	Age ⟶ Metabolic ⟶ Tobacco	12.51	4	2.274	6	0.133	Age–Diabetes; Age–Overweight/Obesity; Age–Tobacco; Diabetes–Overweight/Obesity; Diabetes–Tobacco; Overweight/Obesity–Tobacco
M3	Age ⟶ Behavioral ⟶ Tobacco	12.56	4	2.376	6	0.133	Age–Alcohol; Age–Diabetes; Age–Tobacco; Alcohol–Diabetes; Alcohol–Tobacco; Diabetes–Tobacco
M4	Age ⟶ Lipid ⟶ Tobacco	12.62	4	2.288	6	0.133	Age–Cholesterol; Age–Diabetes; Age–Tobacco; Cholesterol–Diabetes; Cholesterol–Tobacco; Diabetes–Tobacco
M5	Age ⟶ Genetic ⟶ Tobacco	13.81	4	2.407	6	0.133	Age–Diabetes; Age–Family history; Age–Tobacco; Diabetes–Family history; Diabetes–Tobacco; Family history–Tobacco
M6	Age ⟶ Diabetes backbone + social node	≥ 12.21	5	3.309	10	0.222	Age–Diabetes; Age–Tobacco; Age–Marital status; Age–Alcohol; Diabetes–Tobacco; Diabetes–Marital status; Diabetes–Alcohol; Tobacco–Marital status; Tobacco–Alcohol; Marital status–Alcohol
M7	Age ⟶ Diabetes backbone + lipid	≥ 12.21	5	3.221	10	0.222	Age–Diabetes; Age–Tobacco; Age–Marital status; Age–Cholesterol; Diabetes–Tobacco; Diabetes–Marital status; Diabetes–Cholesterol; Tobacco–Marital status; Tobacco–Cholesterol; Marital status–Cholesterol
M8	Age ⟶ Metabolic cluster	≥ 12.21	5	3.207	10	0.222	Age–Diabetes; Age–Tobacco; Age–Marital status; Age–Overweight/Obesity; Diabetes–Tobacco; Diabetes–Marital status; Diabetes–Overweight/Obesity; Tobacco–Marital status; Tobacco–Overweight/Obesity; Marital status–Overweight/Obesity
M9	Age ⟶ Family susceptibility	≥ 12.21	5	3.340	10	0.222	Age–Diabetes; Age–Tobacco; Age–Marital status; Age–Family history; Diabetes–Tobacco; Diabetes–Marital status; Diabetes–Family history; Tobacco–Marital status; Tobacco–Family history; Marital status–Family history
M10	Behavioral–lipid path	≥ 12.21	5	2.845	10	0.222	Age–Diabetes; Age–Tobacco; Age–Alcohol; Age–Cholesterol; Diabetes–Tobacco; Diabetes–Alcohol; Diabetes–Cholesterol; Tobacco–Alcohol; Tobacco–Cholesterol; Alcohol–Cholesterol
M11	Behavioral–adiposity path	≥ 12.21	5	2.831	10	0.222	Age–Diabetes; Age–Tobacco; Age–Alcohol; Age–Overweight/Obesity; Diabetes–Tobacco; Diabetes–Alcohol; Diabetes–Overweight/Obesity; Tobacco–Alcohol; Tobacco–Overweight/Obesity; Alcohol–Overweight/Obesity
M12	Behavioral–genetic path	≥ 12.21	5	2.964	10	0.222	Age–Diabetes; Age–Tobacco; Age–Alcohol; Age–Family history; Diabetes–Tobacco; Diabetes–Alcohol; Diabetes–Family history; Tobacco–Alcohol; Tobacco–Family history; Alcohol–Family history
M13	Lipid–adiposity interaction	≥ 12.21	5	2.743	10	0.222	Age–Diabetes; Age–Tobacco; Age–Cholesterol; Age–Overweight/Obesity; Diabetes–Tobacco; Diabetes–Cholesterol; Diabetes–Overweight/Obesity; Tobacco–Cholesterol; Tobacco–Overweight/Obesity; Cholesterol–Overweight/Obesity
M14	Lipid–genetic interaction	≥ 12.21	5	2.876	10	0.222	Age–Diabetes; Age–Tobacco; Age–Cholesterol; Age–Family history; Diabetes–Tobacco; Diabetes–Cholesterol; Diabetes–Family history; Tobacco–Cholesterol; Tobacco–Family history; Cholesterol–Family history
M15	Adiposity–genetic interaction	≥ 12.21	5	2.862	10	0.222	Age–Diabetes; Age–Tobacco; Age–Overweight/Obesity; Age–Family history; Diabetes–Tobacco; Diabetes–Overweight/Obesity; Diabetes–Family history; Tobacco–Overweight/Obesity; Tobacco–Family history; Overweight/Obesity–Family history
M16	Education‐mediated behavioral path	≥ 12.21	5	2.626	10	0.222	Age–Diabetes; Age–Tobacco; Age–Education; Age–Alcohol; Diabetes–Tobacco; Diabetes–Education; Diabetes–Alcohol; Tobacco–Education; Tobacco–Alcohol; Education–Alcohol
M17	Education‐mediated lipid path	≥ 12.21	5	2.538	10	0.222	Age–Diabetes; Age–Tobacco; Age–Education; Age–Cholesterol; Diabetes–Tobacco; Diabetes–Education; Diabetes–Cholesterol; Tobacco–Education; Tobacco–Cholesterol; Education–Cholesterol
M18	Education‐mediated metabolic path	≥ 12.21	5	2.524	10	0.222	Age–Diabetes; Age–Tobacco; Age–Education; Age–Overweight/Obesity; Diabetes–Tobacco; Diabetes–Education; Diabetes–Overweight/Obesity; Tobacco–Education; Tobacco–Overweight/Obesity; Education–Overweight/Obesity
M19	Education‐mediated genetic path	≥ 12.21	5	2.657	10	0.222	Age–Diabetes; Age–Tobacco; Age–Education; Age–Family history; Diabetes–Tobacco; Diabetes–Education; Diabetes–Family history; Tobacco–Education; Tobacco–Family history; Education–Family history
M20	Education‐mediated social path	≥ 12.21	5	3.002	10	0.222	Age–Diabetes; Age–Tobacco; Age–Education; Age–Marital status; Diabetes–Tobacco; Diabetes–Education; Diabetes–Marital status; Tobacco–Education; Tobacco–Marital status; Education–Marital status

**FIGURE 3:**
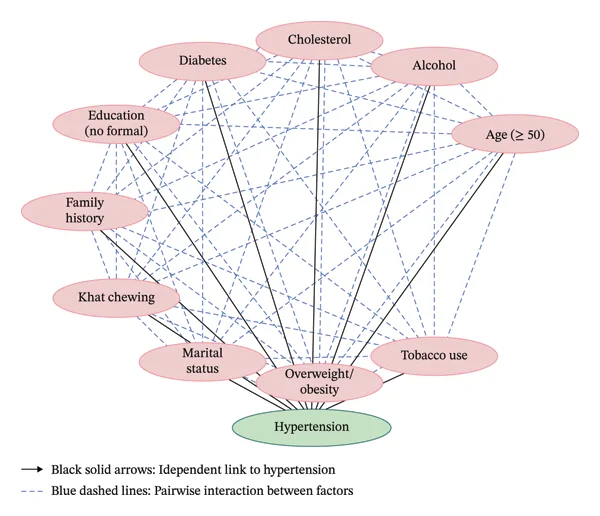
Dicyclic diagram showing hypothetical interactions among hypertension predictors.

#### 3.5.10. Certainty of Evidence

The certainty of the evidence was assessed using the GRADE framework. Evidence from observational studies was initially rated as low and downgraded when the heterogeneity was substantial or the estimates were imprecise. Moderate certainty was observed for age, diabetes, adiposity, and tobacco exposure, reflecting relatively consistent associations and large effect estimates. In contrast, education and khat chewing were rated as having very low certainty owing to substantial heterogeneity, imprecision, and potential publication biases. Other social and behavioral factors generally remained uncertain, reflecting the variability in findings across studies. A detailed summary of the GRADE assessments is provided in Supporting File 4.

## 4. Discussion

This review synthesizes evidence on the factors associated with hypertension in Ethiopia, demonstrating that the risk is distributed across demographic, behavioral, dietary, socioeconomic, and metabolic domains. The findings indicate that hypertension is better understood as arising from the accumulation and co‐occurrence of multiple risk factors rather than from isolated exposures. Although several factors have shown consistent associations, the substantial heterogeneity across studies highlights the complexity of hypertension epidemiology in low‐and middle‐income settings [1, 5].

### 4.1. Demographic Determinants and Structural Patterns

Increasing age showed the most consistent association with hypertension across studies, with both categorical and continuous analyses indicating an increased risk of hypertension with age. This aligns with the established evidence linking vascular aging, endothelial dysfunction, and cumulative exposure to risk factors with elevated blood pressure [2, 3]. In the Ethiopian context, age may reflect prolonged exposure to unaddressed risk factors and delayed access to screening. Sex showed inconsistent associations, suggesting that the observed differences may reflect behavioral and contextual exposures rather than biological effects alone. Similar patterns have been reported in African populations, where sex differences attenuate after adjusting for lifestyle and metabolic factors [28]. Residence also demonstrated variable associations, indicating that urbanization likely operates through underlying behavioral and environmental changes rather than as an independent determinant [5, 9].

### 4.2. Socioeconomic and Psychosocial Factors

Socioeconomic factors, including education, income, and occupation, are generally associated with hypertension; however, the results are heterogeneous. These results support the social determinants perspective, in which structural conditions shape exposure to behavioral and metabolic risks [1, 11]. Education and awareness of hypertension were associated with risk, suggesting that health literacy and access to information may influence prevention and early detection of hypertension. However, the certainty of evidence for these factors was generally low to very low, reflecting heterogeneity, imprecision, and, in some cases, potential publication bias. [32]. Therefore, these findings should be interpreted as context‐dependent associations rather than as stable determinants.

### 4.3. Behavioral Exposures and Lifestyle Factors

Behavioral factors, including alcohol and tobacco use, khat chewing, physical inactivity, and sleep duration, showed variable but generally positive associations with hypertension. The observed heterogeneity likely reflects differences in exposure, population characteristics, and cultural practices. These findings are consistent with broader evidence linking such exposure to hypertension through mechanisms, including sympathetic activation, metabolic dysregulation, and vascular dysfunction [73–75]. However, given the variability across studies and the predominance of cross‐sectional designs, these associations should be interpreted with caution.

### 4.4. Dietary Factors and Cardiometabolic Risk

Dietary factors, particularly high salt intake, are consistently associated with the development of hypertension. This aligns with the strong experimental and epidemiological evidence linking sodium intake to increased vascular resistance and elevated blood pressure [10]. Other dietary factors, including overall dietary patterns and fruit and vegetable intake, have shown mixed associations, likely reflecting differences in measurements and cultural dietary practices. Nonetheless, global evidence supports the protective role of plant‐based diets and the adverse effects of high‐fat and processed foods [76].

### 4.5. Metabolic Factors and Certainty of Evidence

Metabolic factors showed the most consistent and strongest associations with hypertension. Obesity, central adiposity, diabetes, and high cholesterol levels were all associated with increased odds of hypertension across studies, consistent with established cardiometabolic pathways linking adiposity, insulin resistance, and dyslipidemia to elevated blood pressure [6, 10]. The GRADE assessment indicated moderate certainty of evidence for these factors, reflecting relatively consistent associations and larger effect sizes, despite heterogeneity [31]. In contrast, behavioral and socioeconomic factors were generally rated as having low certainty owing to the variability in the findings and methodological limitations.

### 4.6. Framework‐Based Synthesis and Interaction Patterns

An exploratory framework incorporating risk responsiveness (Ri), GTT, and CRW was applied to support comparative interpretation. These metrics indicated that demographic and metabolic factors tended to show stronger and more consistent associations, whereas behavioral and socioeconomic factors were more variable. The analysis of cumulative patterns suggested that relatively small combinations of factors may account for higher levels of observed risk, with recurring involvement of age and metabolic indicators and more variable inclusion of behavioral and social factors. This pattern aligns with multicausal epidemiological frameworks, in which diseases arise from combinations of component causes rather than from a single exposure [12]. However, these findings are exploratory and should not be interpreted as evidence of causal pathways, particularly given the observational nature of the studies included.

### 4.7. Limitations

This study has several limitations. The predominance of cross‐sectional studies limits causal inference and may contribute to observed heterogeneity. Importantly, because the included studies varied in baseline hypertension prevalence, population characteristics, exposure definitions, measurement approaches, and covariate adjustment sets, pooled effect estimates were not treated as directly exchangeable measures of absolute risk. Therefore, the quantitative framework was used as an exploratory approach to facilitate the comparative interpretation of the magnitude and consistency of the observed associations rather than to make direct causal or absolute‐risk comparisons across factors. Variations in exposure definitions, measurement approaches, and analytical adjustments constitute a limitation of this review’s outcome despite attempts at data harmonization following the GBD technique. Some analyses were based on a small number of studies, which limited the precision and ability to assess publication bias. Although the methodological quality was generally acceptable, the certainty of evidence varied across factors, with many associations rated as low owing to inconsistency and imprecision [31].

### 4.8. Implications for Policy and Future Research

These findings support the need for integrated hypertension prevention strategies that address both metabolic and behavioral determinants of hypertension. Screening efforts should prioritize individuals at higher risk, including older adults and those with metabolic conditions, whereas population‐level interventions should focus on modifiable exposures, such as salt reduction, tobacco control, and physical activity promotion [10, 73]. Future research should prioritize longitudinal cohort designs, improved exposure measurements, and standardized reporting to strengthen these causal inferences. Broader geographic representation and comparative analyses across settings would further enhance our understanding of the contextual variations in hypertension risk factors.

## 5. Conclusion

This review shows that hypertension in Ethiopia is associated with interacting demographic, metabolic, behavioral, and socioeconomic factors rather than isolated risk factors. Metabolic factors, particularly adiposity and diabetes, showed the most consistent associations, whereas behavioral and social factors showed greater variability. These findings support a multilevel approach to hypertension prevention that combines targeted screening and population‐level strategies. However, the predominance of observational evidence highlights the need for longitudinal studies to strengthen the causal understanding and inform policies.

## Author Contributions

Conceptualization and methodology: Martins Nweke and Julian D. Pillay. Literature search and study selection: Martins Nweke and a librarian. The underlying data were accessed and verified by Martins Nweke and research assistants, and data analyses and visualizations were performed by Martins Nweke and a research assistant. Writing: Martins Nweke and a research assistant. Editing and Review: Martins Nweke and Julian D. Pillay. Approval: Martins Nweke & Julian D. Pillay.

## Funding

The study was funded by the Heart and Stroke Foundation South Africa (HSFSA): Grant ID: HSFSAJDPGRANT2024.

## Consent

This study was not applicable because the data were derived from articles published in public domains. Individual participant data were excluded.

## Conflicts of Interest

The authors declare no conflicts of interest.

## Supporting Information

Additional supporting information can be found online in the Supporting Information section.

## Supporting information


**Supporting Information 1** Supporting File 1: Search Strategy.

**Supporting Information 2** Supporting File 2: Risk of bias assessment.

**Supporting Information 3** Supporting File 3: Results of individual studies.

**Supporting Information 4** Supporting File 4: Certainty of evidence using GRADE framework.

## Data Availability

This study employed an evidence synthesis approach and did not involve individual patient data. All data extracted for this systematic review and used for the meta‐analyses are provided in Tables and Supporting Files.

## References

[1] Aravkin A. Y. , Zheng P. , Abbafati C. , Abbas K. M. , Abbasi-Kangevari M. et al., Global Burden of 87 Risk Factors in 204 Countries and Territories, 1990–2019, Lancet. (2020) 396, 1223–1249, 10.1016/S0140-6736(20)30752-2. PMC7566194 33069327

[2] Carrillo-Larco R. M. , Danaei G. , Riley L. M. , Paciorek C. J. , Stevens G. A. et al., Worldwide Trends in Hypertension Prevalence and Control from 1990 to 2019, Lancet. (2021) 398, 957–980, 10.1016/S0140-6736(21)01330-1. PMC8446938 34450083

[3] World Health Organization (Who) , Hypertension Fact Sheet, 2023, WHO, Geneva.

[4] Forouzanfar M. H. , Liu P. , Roth G. A. et al., Global Burden of Hypertension and Systolic Blood Pressure ≥110–115 mmHg, JAMA. (2017) 317, no. 2, 10.1001/jama.2016.19043. 28097354

[5] Roth G. A. , Mensah G. A. , Johnson C. O. et al., Global Burden of Cardiovascular Diseases and Risk Factors, 1990–2019, Journal of the American College of Cardiology. (2020) 76, no. 25, 2982–3021, 10.1016/j.jacc.2020.11.010. PMC7755038 33309175

[6] Mills K. T. , Stefanescu A. , and He J. , Global Epidemiology of Hypertension, Nature Reviews Nephrology. (2020) 16, no. 4, 223–237, 10.1038/s41581-019-0244-2. PMC7998524 32024986

[7] Ibrahim M. M. and Damasceno A. , Hypertension in Developing Countries, Lancet. (2012) 380, no. 9841, 611–619, 10.1016/S0140-6736(12)60861-7. 22883510

[8] Adeloye D. , Basquill C. , Aderemi A. V. , Thompson J. Y. , and Obi F. A. , Prevalence of Hypertension in Nigeria, Journal of Hypertension. (2015) 33, no. 2, 230–242, 10.1097/HJH.0000000000000413. 25380154

[9] Ataklte F. , Erqou S. , Kaptoge S. , Taye B. , Echouffo-Tcheugui J. B. , and Kengne A. P. , Undiagnosed Hypertension in sub-Saharan Africa, Hypertension. (2015) 65, no. 2, 291–298, 10.1161/HYPERTENSIONAHA.114.04394. 25385758

[10] He F. J. , Li J. , and MacGregor G. A. , Salt Reduction and Blood Pressure, BMJ. (2013) 346, 10.1136/bmj.f1325. 23558162

[11] Marmot M. , Friel S. , Bell R. , Houweling T. A. , and Taylor S. , Social Determinants of Health, Lancet. (2008) 372, 1661–1669, 10.1016/S0140-6736(08)61690-6. 18994664

[12] Susser M. , Causal Thinking in the Health Sciences, Journal of Epidemiology & Community Health. (2001) 55, 376–378.

[13] Moola S. , Munn Z. , Tufunaru C. et al., Systematic Reviews of Etiology and Risk, 2024, JBI Manual for Evidence Synthesis.

[14] Australian Institute of Health and Welfare , Cancer in Australia: An Overview, 2012, AIHW, Canberra.

[15] Tomlinson J. and Moonesinghe S. , Risk Assessment in Anesthesia, Anesthesiology and Intensive Care Medicine. (2016) 17, no. 10, 486–491, 10.1016/j.mpaic.2016.07.003.

[16] Ladouceur M. , Waldmann V. , Bartoletti S. , Chaix M. A. , and Khairy P. , Ventricular Arrhythmia in Congenital Heart Disease, International Journal Cardiology. (2023) 13. 10.1016/j.ijcchd.2023.100463 PMC11657340 39712230

[17] Rothman K. J. , Causes, American Journal of Epidemiology. (1976) 104, no. 6, 587–592, 10.1093/oxfordjournals.aje.a112335. 998606

[18] Hill A. B. , The Environment and Disease: Association or Causation?, Proceedings of the Royal Society of Medicine. (1965) 58, no. 5, 295–300, 10.1177/003591576505800503. PMC1898525 14283879

[19] Stanaway J. D. , Afshin A. , Gakidou E. et al., Comparative Risk Assessment of 84 Risks, Lancet. (2018) 392, 1923–1994, 10.1016/S0140-6736(18)32225-6. PMC6227755 30496105

[20] Yusuf S. , Hawken S. , Ounpuu S. et al., INTERHEART Study, Lancet. (2004) 364, 937–952, 10.1016/S0140-6736(04)17018-9. 15364185

[21] Page M. J. , McKenzie J. E. , Bossuyt P. M. et al., PRISMA 2020 Statement, BMJ. (2021) 10.1136/bmj.n71.

[22] Munn Z. , Stern C. , Aromataris E. , Lockwood C. , and Jordan Z. , Typology of Systematic Reviews, BMC Medical Research Methodology. (2018) 18, 10.1186/s12874-017-0468-4. PMC5761190 29316881

[23] Centers for Disease Control and Prevention (Cdc) , Analyzing and Interpreting Data, Field Epidemiology Manual. (2024) Atlanta, GA.

[24] Szumilas M. , Explaining Odds Ratios, Journal of the Canadian Academy of Child and Adolescent Psychiatry. (2010) 19, 227–229. PMC2938757 20842279

[25] Andhavarapu S. , Yardi I. , Bzhilyanskaya V. et al., Post-Traumatic Stress Among Healthcare Workers, Psychiatry Research. (2022) 317, 10.1016/j.psychres.2022.114890. PMC9573911 36260970

[26] Nweke M. , Govender N. , and Pillay J. , Risk Stratification of Hypertension in South Africa, Frontiers in Cardiovascular Medicine. (2026) 13, 10.3389/fcvm.2026.1710798. PMC12967931 41808750

[27] Nweke M. , Oyirinnaya P. , Nwoha P. et al., Afrocentric Predictive Model for Stroke, BMC Neurology. (2025) 25, 10.1186/s12883-025-04229-x. PMC12232583 40624481

[28] Nwagha T. and Nweke M. , Stratification of Risk Factors for Lung cancer-associated Venous Thromboembolism, Clinical Medicine Insights: Oncology. (2023) 17. 10.1177/11795549231175221 PMC10328178 37426681

[29] Nweke M. , Ibeneme S. , Pillay J. , and Mshunqane N. , Characterization and Risk Stratification of Coronary Artery Disease in People Living with HIV, Frontiers in Cardiovascular Medicine. (2025) . 10.3389/fcvm.2025.1586019 PMC12378808 40873612

[30] Ibeneme S. , Odoh E. , Nweke M. , and Ibeneme G. , HIV-specific Fall Risk Prediction Model, BMC Infectious Diseases. (2024) 24. 10.1186/s12879-024-10141-5 PMC11653889 39696054

[31] Birhan T. A. , Molla M. D. , Abdulkadir M. , and Tesfa K. H. , ACE Gene Polymorphism and Hypertension Risk in Ethiopia, PLoS One. (2022) 17, 10.1371/journal.pone.0276021. PMC9648817 36355860

[32] Page M. J. , Sterne J. A. C. , Boutron I. et al., ROB-ME: a Tool for Assessing the Risk of Bias due to Missing Evidence in Systematic Reviews with meta-analysis, BMJ. (2023) 383, 10.1136/bmj-2023-076754. 37984978

[33] Essa E. , Shitie D. , Yirsaw M. T. , and Wale M. Z. , Undiagnosed Hypertension and Associated Factors Among Adults in Debre Markos Town, Northwest Ethiopia: a Community-based cross-sectional Study, SAGE Open Medicine. (2022) 10, 10.1177/20503121221094223. PMC9087240 35558191

[34] Dereje N. , Earsido A. , Temam L. , and Abebe A. , Uncovering the High Burden of Hypertension and Its Predictors Among Adults in Hosanna Town, Southern Ethiopia: a Community-based cross-sectional Study, BMJ Open. (2020) 10, 10.1136/bmjopen-2019-035823. PMC7554456 33051227

[35] Endrias E. E. , Mamito T. T. , Hardido T. G. , and Ataro B. A. , Prevalence of prehypertension/hypertension and Its Associated Factors Among Adults in the Wolaita Zone of Southern Ethiopia: a cross-sectional Study, Inquiry. (2024) 61, 10.1177/00469580241246968. PMC11032063 38641978

[36] Gebremedhin M. H. , Lake E. A. , and Gebrekirstos L. G. , Heavy Khat Chewing and Dyslipidemia as Modifiable Risk Factors for Hypertension Among Patients in Southwest Ethiopia: an Unmatched case-control Study, PLoS One. (2021) 16, no. 10, 10.1371/journal.pone.0259078. PMC8547649 34699551

[37] Gebremeskel G. G. , Haile T. G. , Gebrewahd G. T. , and Tadesse D. B. , High Blood Pressure and Associated Factors Among Aksum University Students in Northern Ethiopia, The Internet Journal of Public Health. (2024) 69, 10.3389/ijph.2024.1607275. PMC11144848 38832212

[38] Haile T. G. and Geset Haile D. B. , Hypertension-Related Factors Among Adults in Ethiopia, Journal Details not Clearly Specified in the Source. (2021) .

[39] Solela G. , Arega B. , Tewabe E. , Ambachew R. , Kassahun S. , and Legese S. , Prevalence of Newly Diagnosed Hypertension and Associated Factors in Addis Ababa, BMC Cardiovascular Disorders. (2024) 24, 10.1186/s12872-024-04171-9. PMC11401254 39277708

[40] Zekewos A. , Egeno T. , and Loha E. , Magnitude of Hypertension and Its Risk Factors in Southern Ethiopia, PLoS One. (2019) 14, no. 8, 10.1371/journal.pone.0221726. PMC6713347 31461475

[41] Abebe S. M. , Berhane Y. , Worku A. , and Getachew A. , Prevalence and Associated Factors of Hypertension in Northwest Ethiopia, PLoS One. (2015) 10, 10.1371/journal.pone.0125210. PMC4409323 25909382

[42] Abebe S. and Yallew W. W. , Prevalence of HTN Among Adult Outpatient Clients in Addis Ababa, BMC Research Notes. (2019) 12, 10.1186/s13104-019-4127-1. PMC6376725 30764864

[43] Adal A. B. , Kassa R. N. , Habte M. H. et al., Undiagnosed Hypertension Among long-distance Bus Drivers in Addis Ababa, 2024, 10.1371/journal.pone.0292890. PMC10868739 38359046

[44] Anjulo U. , Haile D. , and Wolde A. , Prevalence of Hypertension and Associated Factors in Areka Town, Blood Press Control. (2021) 14, 43–54, 10.2147/IBPC.S295574. PMC7981153 33758539

[45] Asemu M. M. , Yalew A. W. , Kabeta N. D. , and Mekonnen D. , Prevalence and Risk Factors of Hypertension in Addis Ababa, PLoS One. (2021) 16, 10.1371/journal.pone.0248934. PMC8016337 33793641

[46] Asfaw L. S. , Ayanto S. Y. , and Gurmamo F. L. , Hypertension and Its Associated Factors in Hosanna Town, BMC Research Notes. (2018) 11, 10.1186/s13104-018-3435-1. PMC5956548 29769149

[47] Asresahegn H. , Tadesse F. , and Beyene E. , Prevalence and Associated Factors of Hypertension Among Adults in Ethiopia: a Community Based cross-sectional Study, BMC Research Notes. (2017) 10, no. 1, 10.1186/s13104-017-2966-1. PMC5704552 29183367

[48] Awoke A. , Awoke T. , Alemu S. , and Megabiaw B. , Prevalence and Associated Factors of Hypertension in Gondar, BMC Cardiovascular Disorders. (2012) 12, 10.1186/1471-2261-12-113. PMC3519757 23186560

[49] Ayalew T. L. and Wale B. G. , Burden of Undiagnosed Hypertension in Wolaita Sodo Town, BMC Cardiovascular Disorders. (2022) 22, 10.1186/s12872-022-02733-3. PMC9238150 35761173

[50] Badego B. , Yoseph A. , and Astatkie A. , Prevalence and Risk Factors of Hypertension Among Civil Servants in Sidama, PLoS One. (2020) 15, no. 6, 10.1371/journal.pone.0234485. PMC7289366 32525916

[51] Bayray A. , Meles K. G. , and Sibhatu Y. , Magnitude and Risk Factors of Hypertension in Tigray, PLoS One. (2018) 13, 10.1371/journal.pone.0204879. PMC6169912 30281660

[52] Belachew A. , Tewabe T. , Miskir Y. et al., Prevalence of Hypertension Among Adult Patients in Northwest Ethiopia, BMC Research Notes. (2018) 11, 10.1186/s13104-018-3986-1. PMC6288930 30526686

[53] Belay D. G. , Wolde H. F. , Molla M. , Aragie H. , and Adugna D. , Hypertension Among Adult Patients in Wolkait Tegedie Zone, Frontiers in Neurology. (2022) . 10.3389/fneur.2022.943595 PMC9414034 36034276

[54] Berhe D. A. , Yenit M. K. , and Baraki A. G. , Burden of Hypertension Among Adults in Addis Ababa, Vascular Health and Risk Management. (2020) 16, 545–552, 10.2147/VHRM.S285900. PMC7751300 33364773

[55] Bonsa F. , Gudina E. K. , and Hajito K. W. , Prevalence of Hypertension in Bedele Town, Ethiopan Journal Health Science. (2014) 24, 21–26, 10.4314/ejhs.v24i1.3. PMC3929924 24591795

[56] Dejenie M. , Kerie S. , and Reba K. , Undiagnosed Hypertension Among Bank Workers in Bahir Dar, PLoS One. (2021) 16, 10.1371/journal.pone.0252298. PMC8158901 34043717

[57] Feyisa B. R. , Tamiru A. , Debelo S. et al., Hypertension and Obesity in University Employees, BMJ Open. (2023) 13, 10.1136/bmjopen-2022-070656. PMC10347519 37438078

[58] Gedamu D. K. and Sisay W. , Hypertension Among Public Servants in North Wollo, Vascular Health and Risk Management. (2021) 17, 363–370, 10.2147/VHRM.S298138. PMC8232868 34188478

[59] Gedefaw A. , Ayele Y. Y. , Wudie G. , Tefera A. , Yirdaw A. K. , and Demilew T. , Pulmonary Hypertension Among Rheumatic Heart Disease Patients, BMC Cardiovascular Disorders. (2025) 25, 10.1186/s12872-025-04476-3. PMC11734543 39810078

[60] Gelassa F. R. , Birhanu A. , Shibiru A. , Nagari S. L. , and Jabena D. E. , Undiagnosed Hypertension in Rural Ethiopia, PLoS One. (2022) 17, 10.1371/journal.pone.0277709. PMC9754235 36520859

[61] Geleta G. T. , Cheme M. C. , and Roro E. M. , Determinants of Hypertension in the Town of Nekemte, BMC Research Notes. (2019) 12, 10.1186/s13104-019-4804-0. PMC6873673 31753005

[62] Gudina E. K. , Michael Y. , and Assegid S. , Hypertension and Risk Factors in Southwest Ethiopia, Blood Press Control. (2013) 6, 111–117, 10.2147/IBPC.S47298. PMC3753877 23986649

[63] Gutema B. T. , Chuka A. , Ayele G. et al., Obesity Indices and Hypertension in Southern Ethiopia, BMC Cardiovascular Disorders. (2020) 20, 10.1186/s12872-020-01686-9. PMC7507659 32957951

[64] Haile M. , Degelo T. , Adilo T. M. , Adem F. M. , and Gidisa B. , Hypertension Among Adults Living with HIV in Addis Ababa, HIV. (2024) 16, 109–122, 10.2147/HIV.S447396. PMC10963170 38533310

[65] Helelo T. P. , Gelaw Y. A. , and Adane A. A. , Hypertension in Durame Town, PLoS One. (2014) 9, 10.1371/journal.pone.0112790. PMC4240541 25415321

[66] Kassie S. A. , Beyene K. G. , and Tiruneh M. A. , Determinants of Hypertension in Addis Ababa, International Journal Public Health Science. (2020) 9, 121–128.

[67] Kiber M. , Wube M. , Temesgen H. , Woyraw W. , and Belay Y. A. , Hypertension in Debre Markos Town, BMC Research Notes. (2019) 12, 10.1186/s13104-019-4431-9. PMC6631738 31307528

[68] Kumma W. P. , Lindtjørn B. , and Loha E. , Hypertension in Wolaita, PLoS One. (2021) 16, 10.1371/journal.pone.0260403. PMC8673671 34910760

[69] Paulose T. , Nkosi Z. Z. , and Endriyas M. , Hypertension in Hawassa City, PLoS One. (2022) 17, 10.1371/journal.pone.0264679. PMC8887727 35231073

[70] Rike M. E. , Diress M. , Dagnew B. et al., Hypertension Among long-distance Truck Drivers, Blood Press Control. (2022) 15, 67–79, 10.2147/IBPC.S361789. PMC9233485 35761970

[71] Saif-Ur-Rahman K. M. , Chiang C. , Gebremariam L. W. et al., Obesity Indices and Hypertension in Northern Ethiopia, BMJ Open. (2021) 10.1136/bmjopen-2021-050969. PMC8487201 34593500

[72] Tadesse T. and Alemu H. , Hypertension Among University Students in Gondar, 2014, 10.1186/1471-2458-14-937. PMC4168247 25201163

[73] Roerecke M. , Kaczorowski J. , Tobe S. W. , Gmel G. , Hasan O. S. M. , and Rehm J. , The Effect of Reduced Alcohol Consumption on Blood Pressure: a Systematic Review and meta-analysis, The Lancet Public Health. (2017) 2, e108–e120, 10.1016/S2468-2667(17)30003-8. PMC6118407 29253389

[74] Piercy K. L. , Troiano R. P. , Ballard R. M. et al., Physical Activity Guidelines for Americans, JAMA. (2018) 320, no. 19, 10.1001/jama.2018.14854. PMC9582631 30418471

[75] Itani O. , Jike M. , Watanabe N. , and Kaneita Y. , Short Sleep Duration and Health Outcomes: a Systematic Review, meta-analysis, and meta-regression, Sleep Medicine. (2017) 32, 246–256, 10.1016/j.sleep.2016.08.006. 27743803

[76] Appel L. J. , Moore T. J. , Obarzanek E. et al., A Clinical Trial of the Effects of Dietary Patterns on Blood Pressure, New England Journal Medical. (1997) 336, no. 16, 1117–1124, 10.1056/NEJM199704173361601. 9099655

