# Supplementary material for: Factors Associated With Hypertension in Ethiopia: A Systematic Review, Meta‐Analysis, and Quantitative Framework for Comparative Association
Source: Int J Hypertens. 2026 Nov 1;2026:7268884. doi: 10.1155/ijhy/7268884 (PMC13724153; doi:10.1155/ijhy/7268884)
Supplement: Supplementary file 1 [file IJHY-2026-7268884-s001.docx]

**Certainty of Evidence using GRADE Framework**

| **Risk factor** | **Risk of bias** | **Inconsistency (I²)** | **Imprecision** | **Publication bias** | **Upgrade (effect size)** | **Final certainty** |
| --- | --- | --- | --- | --- | --- | --- |
| **Age ≥50** | **No serious concern (mostly low risk studies)** | **Serious (I²=88.6%)** | **None** | **None (p=0.57)** | **Large OR=3.84** | **Moderate** |
| **Alcohol** | **No serious concern** | **Serious (I²=85.2%)** | **None** | **None (p=0.395)** | **None** | **Low** |
| **Cholesterol** | **No serious concern** | **Moderate (I²=54.4%)** | **None** | **None (p=0.918)** | **None** | **Low** |
| **Diabetes** | **No serious concern** | **Moderate (I²=63.5%)** | **None** | **Borderline (p=0.0589)** | **Very large OR=4.08** | **Moderate** |
| **Education** | **Some concern (confounding issues)** | **Very serious (I²=93.0%)** | **CI crosses null** | **Evidence of bias (p=3.37e-05)** | **None** | **Very Low** |
| **Family history** | **No serious concern** | **Serious (I²=84.1%)** | **None** | **Possible bias (p=0.014)** | **Strong OR=2.91** | **Low–Moderate** |
| **Khat chewing** | **Some concern** | **Serious (I²=89.8%)** | **Very serious (wide CI 0.83–3.71)** | **None (p=0.773)** | **None** | **Very Low** |
| **Marital status** | **No serious concern** | **Moderate (I²=64.1%)** | **None** | **Not reported** | **None** | **Low** |
| **Overweight/Obesity** | **No serious concern** | **Moderate (I²=66.1%)** | **None** | **None (p=0.9)** | **Dose-response plausible** | **Moderate** |
| **Tobacco use** | **No serious concern** | **Moderate (I²=52.6%)** | **Some imprecision** | **None (p=0.129)** | **Large OR=3.81** | **Moderate** |
