# Supplementary material for: Factors Associated With Hypertension in Ethiopia: A Systematic Review, Meta‐Analysis, and Quantitative Framework for Comparative Association
Source: Int J Hypertens. 2026 Nov 1;2026:7268884. doi: 10.1155/ijhy/7268884 (PMC13724153; doi:10.1155/ijhy/7268884)
Supplement: Supplementary file 2 [file IJHY-2026-7268884-s002.docx]

**Supplementary File 1: Search Strategy**

| Database | Search Terms | Date of Search | Filters applied? | Number retrieved |
| --- | --- | --- | --- | --- |
| PubMed | ((Risk factor or body fat or body mass index or waist circumference or salt or stress or dyslipidemia or smoking or vegetables or alcohol or particulate matter or heavy matters or pregnancy or anxiety or diabetes or depression or intimate partner violence or urbanization or diet or cholesterol or age or poverty or sex or access to healthcare or obesity or overweight  or socioeconomic status or air pollution or meat or occupation or education or sedentary lifestyle[MeSH Terms]) AND (Hypertension or high blood pressure or blood pressure or elevated blood pressure or raised blood pressure or hypertensive[MeSH Terms])) AND (Tanzania OR Kenya OR Uganda OR Burundi OR Comoros OR Djibouti OR Eritrea OR Ethiopia OR Madagascar OR Malawi OR Mauritius OR Mayotte OR Mozambique OR Réunion OR Rwanda OR Seychelles OR Somalia[MeSH Terms]) | 7th Jan 2026 | No | 4800 |
| Academic Search complete | blood pressure or hypertension or hypertensive or htn or elevated blood pressure (All field)  AND risk factors or contributing factors or predisposing factors or predictor or cause or vulnerability factors or obesity or overweight or fat or obese or unhealthy weight or high bmi or body mass index or waist circumference or alcoholism or alcohol dependence or alcohol abuse or alcoholic or alcohol addiction or age or stress or poverty or low-income or low socioeconomic status or dyslipidemia or hyperlipidemia or hypercholesterolemia or pregnancy or diet or nutrition or food habit or eating habit or lifestyle or food or urbanisation or urban development or diabetes or blood sugar or anxiety or smoking or tobacco or cigarette or nicotine or intimate or intimate partner violence or domestic violence or partner abuse or physical inactivity or physically inactive or sedentary or lack of physical activity or occupation or education or salt intake or sodium intake or vegetable intake or vegetable consumption or folate deficiency or depression or meat or d-dimer or particulate matter or heavy metal or sex or gender or HIV or carbon monoxide or homelessness or marital status or beverages or access to healthcare (All field)  AND Tanzania OR Kenya OR Uganda OR Burundi OR Comoros OR Djibouti OR Eritrea OR Ethiopia OR Madagascar OR Malawi OR Mauritius OR Mayotte OR Mozambique OR Réunion OR Rwanda OR Seychelles OR Somalia Or East Africa (Subject term) | 7th Jan 2026 | No | 4621 |
| Medline | blood pressure or hypertension or hypertensive or htn or elevated blood pressure (All field)  AND risk factors or contributing factors or predisposing factors or predictor or cause or vulnerability factors or obesity or overweight or fat or obese or unhealthy weight or high bmi or body mass index or waist circumference or alcoholism or alcohol dependence or alcohol abuse or alcoholic or alcohol addiction or age or stress or poverty or low-income or low socioeconomic status or dyslipidemia or hyperlipidemia or hypercholesterolemia or pregnancy or diet or nutrition or food habit or eating habit or lifestyle or food or urbanisation or urban development or diabetes or blood sugar or anxiety or smoking or tobacco or cigarette or nicotine or intimate or intimate partner violence or domestic violence or partner abuse or physical inactivity or physically inactive or sedentary or lack of physical activity or occupation or education or salt intake or sodium intake or vegetable intake or vegetable consumption or folate deficiency or depression or meat or d-dimer or particulate matter or heavy metal or sex or gender or HIV or carbon monoxide or homelessness or marital status or beverages or access to healthcare (All field)  AND Tanzania OR Kenya OR Uganda OR Burundi OR Comoros OR Djibouti OR Eritrea OR Ethiopia OR Madagascar OR Malawi OR Mauritius OR Mayotte OR Mozambique OR Réunion OR Rwanda OR Seychelles OR Somalia Or East Africa (Subject term) | 7th Jan 2026 | No | 3294 |
| CINAHL | blood pressure or hypertension or hypertensive or htn or elevated blood pressure (All field)  AND risk factors or contributing factors or predisposing factors or predictor or cause or vulnerability factors or obesity or overweight or fat or obese or unhealthy weight or high bmi or body mass index or waist circumference or alcoholism or alcohol dependence or alcohol abuse or alcoholic or alcohol addiction or age or stress or poverty or low-income or low socioeconomic status or dyslipidemia or hyperlipidemia or hypercholesterolemia or pregnancy or diet or nutrition or food habit or eating habit or lifestyle or food or urbanisation or urban development or diabetes or blood sugar or anxiety or smoking or tobacco or cigarette or nicotine or intimate or intimate partner violence or domestic violence or partner abuse or physical inactivity or physically inactive or sedentary or lack of physical activity or occupation or education or salt intake or sodium intake or vegetable intake or vegetable consumption or folate deficiency or depression or meat or d-dimer or particulate matter or heavy metal or sex or gender or HIV or carbon monoxide or homelessness or marital status or beverages or access to healthcare (All field)  AND Tanzania OR Kenya OR Uganda OR Burundi OR Comoros OR Djibouti OR Eritrea OR Ethiopia OR Madagascar OR Malawi OR Mauritius OR Mayotte OR Mozambique OR Réunion OR Rwanda OR Seychelles OR Somalia Or East Africa (Subject term) | 7th Jan 2026 | No | 1297 |
