# Supplementary material for: Factors Associated With Hypertension in Ethiopia: A Systematic Review, Meta‐Analysis, and Quantitative Framework for Comparative Association
Source: Int J Hypertens. 2026 Nov 1;2026:7268884. doi: 10.1155/ijhy/7268884 (PMC13724153; doi:10.1155/ijhy/7268884)
Supplement: Supplementary file 3 [file IJHY-2026-7268884-s003.docx]

| Study (Author, Year) | Q1 Clear inclusion criteria | Q2 Subjects & setting described | Q3 Exposure measured validly | Q4 Standard outcome measurement | Q5 Confounders identified | Q6 Strategies to address confounders | Q7 Outcomes measured reliably | Q8 Appropriate statistical analysis | Score (Yes/8) | Interpretation |
| --- | --- | --- | --- | --- | --- | --- | --- | --- | --- | --- |
| Abebe et al., 2015 | Yes | Yes | Yes | Yes | Yes | Yes | Yes | Yes | 8/8 | Low risk of bias |
| Abebe & Yallew, 2019 | Yes | Yes | Yes | Yes | Yes | Yes | Yes | Yes | 8/8 | Low risk of bias |
| Adal et al., 2024 | Yes | Yes | Yes | Yes | Yes | Yes | Yes | Yes | 8/8 | Low risk of bias |
| Anjulo et al., 2021 | Yes | Yes | Yes | Yes | Yes | Yes | Yes | Yes | 8/8 | Low risk of bias |
| Asemu et al., 2021 | Yes | Yes | Yes | Yes | Yes | Yes | Yes | Yes | 8/8 | Low risk of bias |
| Asfaw et al., 2018 | Yes | Yes | Yes | Yes | Yes | Yes | Yes | Yes | 8/8 | Low risk of bias |
| Asresahegn et al., 2017 | Yes | Yes | Yes | Yes | Yes | Yes | Yes | Yes | 8/8 | Low risk of bias |
| Awoke et al., 2012 | Yes | Yes | Yes | Yes | Yes | Yes | Yes | Yes | 8/8 | Low risk of bias |
| Ayalew et al., 2022 | Yes | Yes | Yes | Yes | Yes | Yes | Yes | Yes | 8/8 | Low risk of bias |
| Badego et al., 2020 | Yes | Yes | Yes | Yes | Yes | Yes | Yes | Yes | 8/8 | Low risk of bias |
| Bayray et al., 2018 | Yes | Yes | Yes | Yes | Yes | Yes | Yes | Yes | 8/8 | Low risk of bias |
| Belachew et al., 2018 | Yes | Yes | Yes | Yes | Yes | Yes | Yes | Yes | 8/8 | Low risk of bias |
| Belay et al., 2022 | Yes | Yes | Yes | Yes | Yes | Yes | Yes | Yes | **8/8** | Low risk of bias |
| Berhe et al., 2020 | Yes | Yes | Yes | Yes | Yes | Yes | Yes | Yes | **8/8** | Low risk of bias |
| Bonsa et al., 2014 | Yes | Yes | Yes | Yes | Yes | Yes | Yes | Yes | **8/8** | Low risk of bias |
| Dejenie et al., 2021 | Yes | Yes | Yes | Yes | Yes | Yes | Yes | Yes | **8/8** | Low risk of bias |
| Dereje et al., 2020 | Yes | Yes | Yes | Yes | Yes | Yes | Yes | Yes | **8/8** | Low risk of bias |
| Endrias et al., 2024 | No | Yes | No | Yes | No | No | Yes | No | **4** | Moderate |
| Essa et al., 2022 | Yes | Yes | Yes | Yes | No | Yes | Yes | Yes | **7** | Low |
| Feyisa 2023 | YES | YES | YES | YES | NO | YES | YES | YES | **7/8** | Low risk |
| Gedamu & Sisay 2021 | YES | YES | YES | YES | YES | YES | YES | YES | **8/8** | Low risk of bias |
| Gebremeskel 2024 | NO | YES | YES | YES | YES | YES | YES | YES | **7/8** | Low risk of bias |
| Gedefaw 2025 | NO | YES | YES | YES | YES | YES | YES | YES | **7/8** | Low risk |
| Gelassa 2022 | YES | YES | YES | YES | YES | YES | YES | YES | **8/8** | Low risk |
| Geleta 2019 | YES | YES | YES | YES | YES | YES | YES | YES | **8/8** | Low risk |
| Geset Haile 2021 | YES | YES | YES | YES | YES | YES | YES | YES | **8/8** | Low risk |
| Gudina 2013 | YES | YES | YES | YES | YES | YES | YES | YES | **8/8** | Low risk |
| Gutema 2020 | YES | YES | YES | YES | NO | NO | YES | YES | **6/8** | Moderate risk |
| Haile 2024 | YES | YES | YES | YES | YES | YES | YES | YES | **8/8** | Low risk |
| Helelo 2014 | YES | YES | YES | YES | YES | YES | YES | YES | **8/8** | Low risk |
| Kiber 2019 | YES | YES | YES | YES | YES | YES | YES | YES | **8/8** | Low risk |
| Kumma 2021 | NO | YES | YES | YES | YES | YES | YES | YES | **7/8** | Low risk |
| Paulose 2022 | YES | YES | YES | YES | YES | YES | YES | YES | **8/8** | Low risk |
| Rike 2022 | YES | YES | YES | YES | YES | YES | YES | YES | **8/8** | Low risk |
| Saif-Ur-Rahman 2021 | YES | YES | YES | YES | YES | YES | YES | YES | **8/8** | Low risk |
| Solela 2024 | YES | YES | YES | YES | YES | YES | YES | YES | **8/8** | Low risk |
| Tadesse 2014 | YES | YES | YES | YES | YES | YES | YES | YES | **8/8** | Low risk |
| Zekewos 2019 | YES | YES | YES | YES | YES | YES | YES | YES | **8/8** | Low risk |

| **Study** | **Q1** | **Q2** | **Q3** | **Q4** | **Q5** | **Q6** | **Q7** | **Q8** | **Q9** | **Q10** | **Score** | **Interpretation** |
| --- | --- | --- | --- | --- | --- | --- | --- | --- | --- | --- | --- | --- |
| Birhan et al., 2022 | Yes | Yes | Yes | Yes | Yes | Yes | Yes | Yes | Yes | Yes | **10/10** | Low risk of bias |
| Gebremedhin 2021 | YES | YES | YES | YES | YES | YES | YES | YES | YES | YES | **10/10** | Low risk |
| Kassie 2020 | YES | NO | YES | YES | YES | YES | YES | YES | YES | YES | **9/10** | Low risk |
